# Safety profile of FLT3 inhibitors in acute myeloid leukemia: a systematic review and meta-analysis of adverse events

**DOI:** 10.1007/s10238-026-02093-8

**Published:** 2026-02-23

**Authors:** Mario Gaio, Alessia Zinzi, Valerio Liguori, Cecilia Cagnotta, Mario Frasca, Ludovica Vittoria Laino, Francesco Rossi, Annalisa Capuano

**Affiliations:** 1 Department of Life Science, Health, and Health Professions, Link Campus University, Rome, Italy; 2 Campania Regional Centre for Pharmacovigilance and Pharmacoepidemiology, Naples, Italy; 3 Department of Experimental Medicine, University of Campania Luigi Vanvitelli, Via Santa Maria di Costantinopoli, 104, Naples, Italy

**Keywords:** Acute myeloid leukemia, FLT3 inhibitors, Midostaurin, Gilteritinib, Quizartinib, Safety

## Abstract

Acute myeloid leukemia (AML) is the most common acute leukemia in adults. Approximately 30% of patients present alterations in the FMS-like receptor tyrosine kinase 3 (FLT3) gene, which are associated with poor prognosis. FLT3 inhibitors – midostaurin (first-generation), gilteritinib and quizartinib (second-generation) – have been developed to block FLT3 activation. Given the need of optimizing treatment in FLT3-mutated AML, we conducted a systematic review and meta-analysis of randomized controlled trials (RCTs) to compare the safety profiles of FLT3 inhibitors. Following the PRISMA statement, we searched Embase, MEDLINE and Cochrane Library. The Cochrane Risk of Bias Tool for RCTs was used for quality assessment. Of 2132 references, seven RCTs, involving 2409 adult patients, met inclusion criteria: quizartinib and midostaurin in two trials each and gilteritinib in three. The most frequently reported adverse events (AEs) were classified under the System Organ Class (SOC) Blood and lymphatic system disorders (*N* = 5474, 58.4% of them related to FLT3 inhibitors). The most frequently observed non-hematological AEs were gastrointestinal disorders, pyrexia, elevated ALT/AST and headache. FLT3 inhibitors are not associated with a significant increase in the risk of AEs compared to standard treatments. No meaningful differences in AE risk were observed among the three drugs. The only exception was an higher risk of ALT increased with gilteritinib (RR = 2.40, 95% CI: 1.16–4.95). Future studies should stratify safety outcomes by demographic and clinical characteristics and incorporate long-term follow-up for a more comprehensive safety assessment in clinical practice.

**Supplementary Information:**

The online version contains supplementary material available at 10.1007/s10238-026-02093-8.

## Introduction

Acute myeloid leukemia (AML) is the most common acute leukemia in adults with an approximate annual incidence of 4.3 per 100,000 population, totaling over 20,000 new cases in the United States every year [1, 2]. The poor prognosis of AML is determined by age – with a 62% estimated 5-year survival in patients diagnosed under the age of 50 years, 37% survival for patients 50–64 years of age, and 9.4% for patients 65 years and older at diagnosis –, gender, body mass index, baseline white blood cells count bone marrow blast (BMB) cell count at the time of diagnosis [3–5]. According to the most recent knowledge, AML can be classified in distinct subtypes based on mutation profiles [6]. In particular, approximately 30% of AML patients present alterations in the FMS-like receptor tyrosine kinase 3 (FLT3) gene, located on chromosome 13q12 that is a member of class III receptor tyrosine kinase family [7, 8]. Patients with FLT3 mutations, particularly FLT3 internal tandem deduplications (ITD) – FLT3-ITD mutations occur in almost 25% of newly diagnosed cases of AML – have a poor prognosis with an increased risk of relapse [9]. As a result of the FLT3 mutations, the FLT3 receptor is perpetually activated in the absence of the FLT3 ligand, increasing proliferation and decreasing apoptosis [10, 11]. FLT3 activating mutations may involve also the tyrosine kinase domain (TKD); however, FLT3-TKD mutations have not been associated with a consistent prognostic impact [12].

FLT3 inhibitors have been developed to block FLT3 activation; they are subdivided into type I – active against both the FLT3-ITD or TKD – and type II inhibitors – only active against FLT3-ITD. Moreover, they are classified into first and second generation based on their kinase specificity and potency: the first generation FLT3 inhibitors (i.e., midostaurin) and second generation (i.e., quizartinib and gilteritinib). Second-generation FLT3 inhibitors were developed to target FLT3 specifically, and thus are more selective with less-off-target toxicity and higher potency [13].

Despite the availability of multiple FLT3 inhibitors, a direct head-to-head comparison of their safety profiles remains limited.

Given the clinical relevance of optimizing treatment choices for patients with FLT3-mutated AML, a comprehensive evaluation of adverse events associated with different FLT3 inhibitors is warranted. For this reason, we conducted a systematic review and meta-analysis of randomized controlled trials to assess and compare the safety profiles of FLT3 inhibitors used in this patient population.

## Methods

The Preferred Reporting Items for Systematic Reviews and Meta-Analysis (PRISMA) statement has been followed in the present systematic review and meta-analysis [14].

### Study inclusion and exclusion criteria

The review included randomized controlled trials (RCTs) assessing the adverse events of any FMS-like tyrosine kinase 3 (FLT3) inhibitor (i.e., gilteritinib, quizartinib, and midostaurin) in adult patients with any type of acute myeloid leukemia (AML). Comparators could include either standard care, another experimental comparator or placebo. Notably, sorafenib was excluded from the analysis as it is approved for indications other than AML (i.e., hepatocellular carcinoma and advanced renal cell carcinoma) and is used off-label only in AML patients with FLT3-ITD mutation after allogeneic stem cell transplantation.

Studies were excluded based on several factors. Studies focusing on populations with clinical characteristics distinct from the target group of adult AML patients with FLT3 mutations were excluded. Studies that did not report on adverse events or lacked adequate data on safety were excluded, as they did not align with the aim of the review.

### Search strategy and study selection

We conducted a systematic search across the following electronic bibliographic databases: Embase, MEDLINE (via PubMed), and the Cochrane Library to identify relevant articles. The search query built for PubMed combined three components: one focused on AML, one on the treatments of interest, and one on the study design (Supplementary Table 1). This query was then adapted for searches in EMBASE and the Cochrane Library. The search was conducted from the inception of each database until December 2024, with no language restrictions applied. Two independent reviewers assessed the studies to determine their eligibility for further evaluation, and any discrepancies were resolved by a third reviewer.

### Data extraction and quality assessment

Data were extracted onto standardized and piloted forms, including information on treatment-related adverse events (AEs). When available, AEs were categorized based on their severity grade. The Cochrane Risk of Bias Tool for RCTs was used for quality assessment [15]. The quality of the included studies was assessed by two independent reviewers and any discrepancies in assessment were resolved through consultation with a third reviewer (Supplementary Fig. 1, Supplementary Fig. 2). Data extraction and the risk of bias assessment were based on information from published studies and protocols and undertaken by one reviewer with a second reviewer checking.

### Statistical analysis

We described and tabulated the adverse events (AEs) reported in each study, both for all AEs and specifically for those classified as Grade 3 and above, as defined by the National Cancer Institute Common Toxicity Criteria (CTC). We then calculated the risk ratio (RR) with a 95% confidence interval (95% CI) for these AEs in each study and pooled the results in a meta-analysis to compare the treatments. A p-value of less than 0.05 was considered statistically significant.

To assess heterogeneity in treatment effects, we used χ² and I² statistics. Heterogeneity was not statistically significant when I^2^ < 50%. A fixed-effect model was applied when heterogeneity was low (I^2^ < 50%), while a random-effects model was used in cases of significant heterogeneity (I^2^ ≥ 50%). Additionally, we conducted a subgroup analysis to investigate the risk of AEs based on different FLT3 mutations, disease stage, age, and sex, when the relevant data were available.

All statistical analysis was performed using R Statistical Software (version 4.4.3; R Foundation for Statistical Computing, Wien, Austria).

## Results

### Search results

**Fig. 1:**
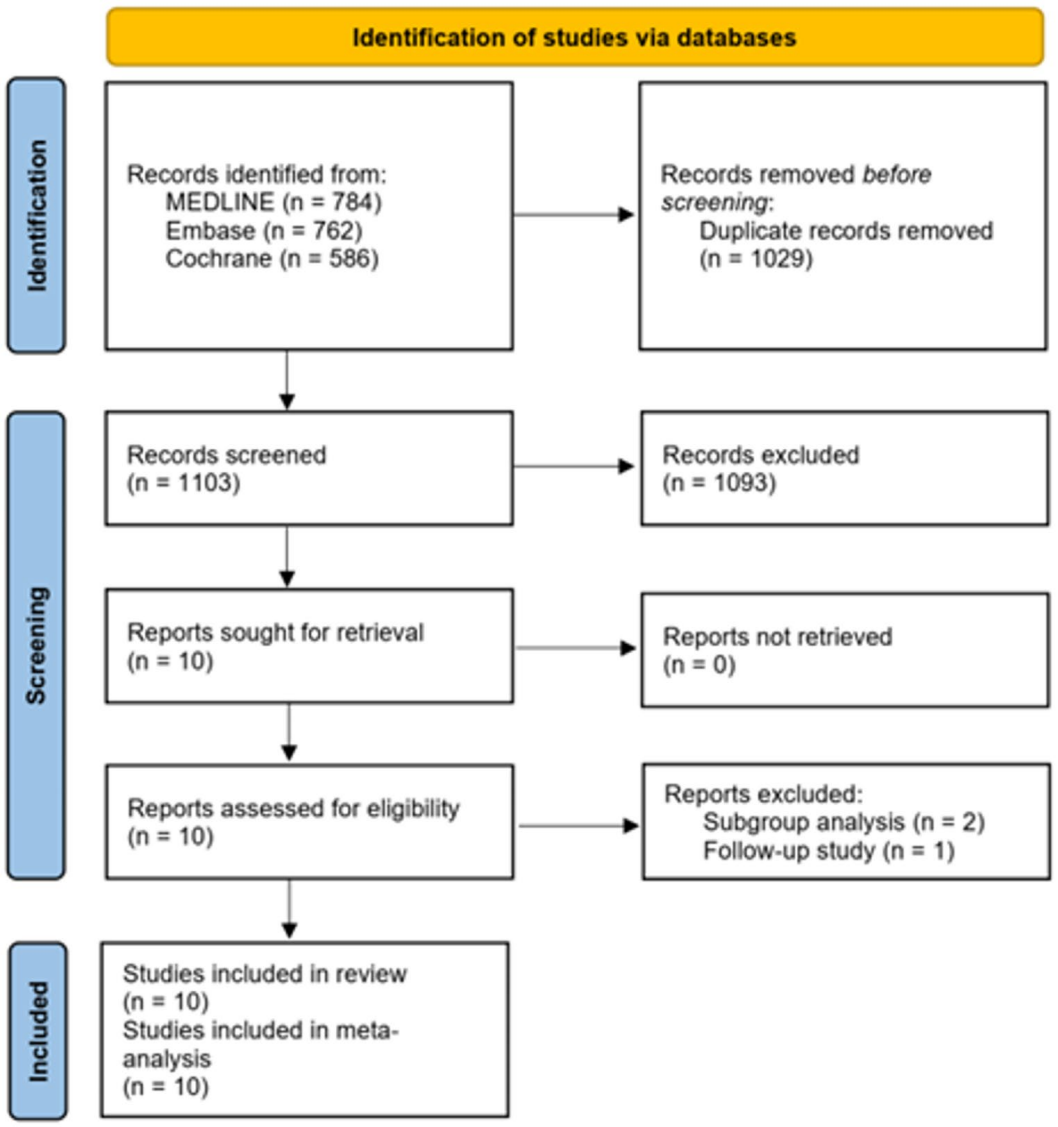
PRISMA flow diagram of study selection process

The database search identified a total of 2132 references. After screening, seven publications reporting on seven completed randomized controlled trials (RCTs) involving 2409 patients met the inclusion criteria for this review [16–22]. All of these studies were included in our meta-analysis (Fig. 1). During the screening process, a significant number of studies were excluded due to their failure to meet the inclusion criteria regarding the population or the data on adverse events.

### Trial characteristics

**Fig. 2:**
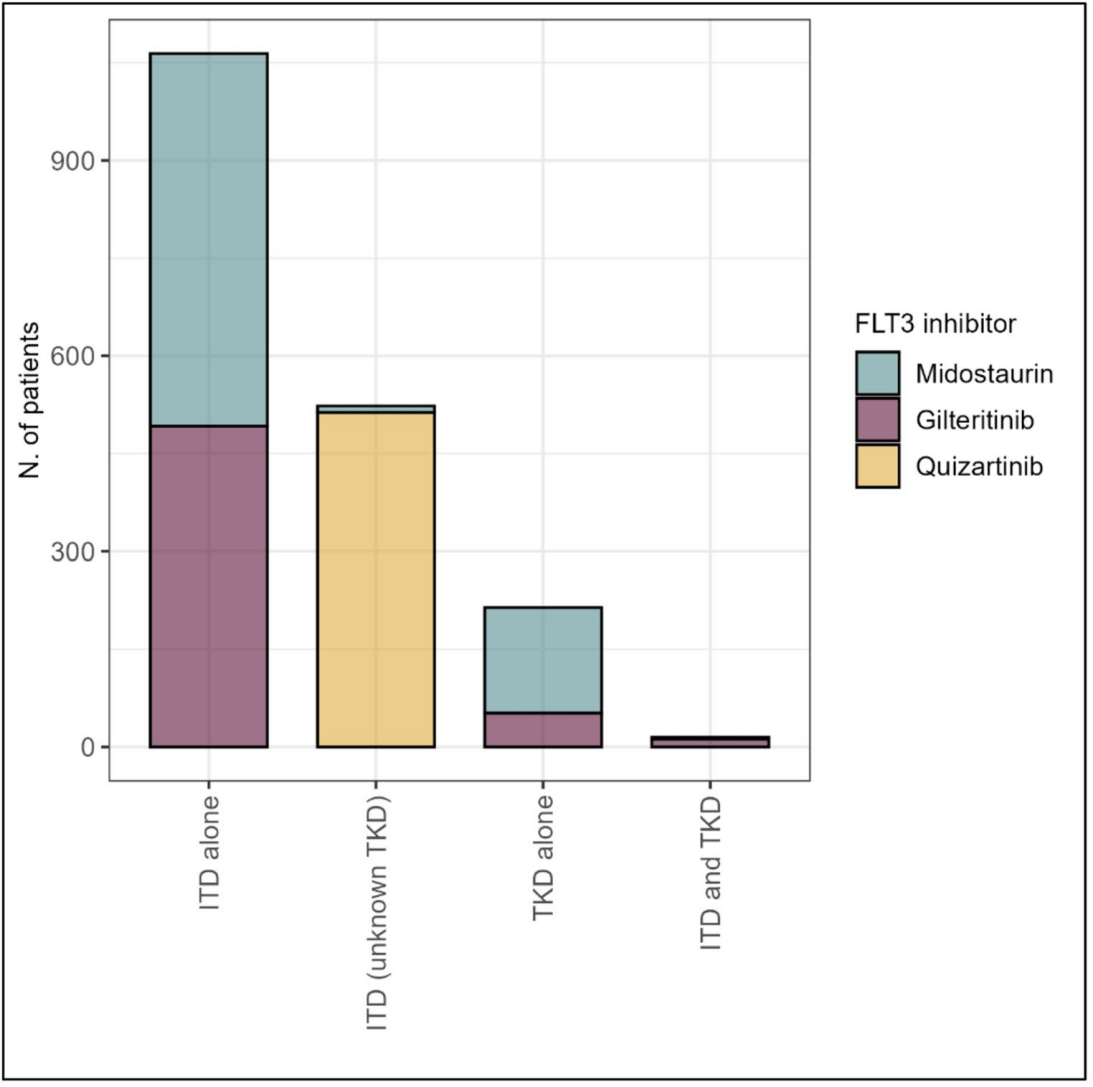
Frequency of AML patients with different FLT3 mutation statuses (FLT3-ITD only, FLT3-TKD only, and FLT3-ITD + TKD mutations), stratified by the specific FLT3 inhibitor used in each trial

All seven trials were multicenter studies investigating three different FLT3 inhibitors: quizartinib in two trials [16, 17], midostaurin in two trials [18, 20], and gilteritinib in three trials [19, 21, 22]. Four trials enrolled adult patients aged 18 and older without an upper age limit [16, 19, 21, 22], while three trials had age restrictions of 75 years old [17], 70 years old [18], and 59 years [20]. Two trials included only patients with newly diagnosed primary AML [17, 20], whereas five trials included patients with either primary AML or AML secondary to myelodysplastic syndromes [16, 18, 19, 21, 22]. Additionally, three trials focused exclusively on patients with FLT3 internal tandem duplication (FLT3-ITD) mutations [16–18] while four included patients with either FLT3-ITD and/or FLT3 tyrosine kinase domain (TKD) point mutations [19–22]. Figure 2 illustrates the frequency of AML patients with FLT3-ITD only, FLT3-TKD only, and FLT3-ITD + TKD mutations, stratified by the specific FLT3 inhibitor used in each trial.

The median age of patients receiving FLT3 inhibitors ranged from 48 years [18] to 78 years [21]. In most trials, female patients outnumbered male patients [16, 17, 19, 20, 22]. Nearly all trials assessed and reported cytogenetic risk status, with the majority of patients classified as having an intermediate risk profile. The main characteristics of the trials included in the systematic review are summarized in Table 1.

**Table 1:** Characteristics of included trials

Author, year of publication	FLT3 inhibitor group	Control group	Inclusion/exclusion criteria	Intervenention baseline median age	Control baseline median age	Female sex	Type of AML	FLT3 mutational status	Cytogenic risk status
Cortes, 2019 [16]	Quizartinib (*N* = 245)	Investigator’s choice of salvage chemotherapy (*N* = 122)	Patients aged ≥ 18 years with ECOG performance status 0–2 with FLT3-ITD primary AML or AML secondary to myelodysplastic syndromes, refractory to or relapsed after at least one cycle of a standard AML therapy (e.g., anthracycline-containing or mitoxantrone-containing AML therapy), with or without allogeneic haemopoietic stem cell transplant.	55.0 (IQR: 46.0–65.0)	57.5 (IQR: 44.0–66.0)	51.8%	NA	ITD (100%)	Favourable (5.4%), Intermediate (74.1%), Unfavourable (10.1%)
Erba, 2023 [17]	Quizartinib + chemoterapy (*N* = 268)	SOC therapy + placebo (*N* = 271)	Patients aged 18–75 years with primary newly diagnosed AML, or AML secondary to myelodysplastic syndromes or a myeloproliferative neoplasm, with an FLT3-ITD mutation (variant allelic frequency ≥ 3%), with an ECOG status of 0–2, and able to receive standard induction chemotherapy. Patients diagnosed with acute promyelocytic leukemia with t(15;17), BCR-ABL1-positive leukemia, or AML secondary to previous chemotherapy or radiotherapy were excluded.	56.0 (IQR: 44.5–65.0)	56.0 (IQR: 47.0–64.0)	54.5%	De novo (92.4%), Secondary (7.6%)	ITD (100%)	Favourable (6.1%), Intermediate (72.3%), Unfavourable (8.5%)
Maziarz, 2020 [18]	Midostaurin (*N* = 30)	SOC therapy (*N* = 30)	Patients aged 18–70 years with FLT3-ITD AML who had undergone a protocol-specified conditioning regimen before hematopoietic stem cell transplantation in first complete remission (following hematologic recovery, transfusion independence, and controlled graft-vs-host disease.	48 (range: 20–61)	56 (range: 20–68)	43.3%	De novo (95.0%), Secondary (5.0%)	ITD only (91.7%), ITD + TKD (8.3)	NA
Perl, 2019 [19]	Gilteritinib (*N* = 245)	Investigator’s choice of salvage chemotherapy (*N* = 124)	Patients aged ≥ 18 years with refractory to one or two cycles of conventional anthracycline-containing induction therapy or with hematologic relapse after a complete remission. FLT3 mutations (ITD and/or TKD) were considered to be present if the mutant-to-nonmutant allelic ratio was at least 0.05.	62.0 (range: 20.0–84.0)	61.5 (range: 19.0–85.0)	54.2%	NA	ITD only (88.4%), TKD only (8.4%), ITD and TKD (1.9%)	Favourable (1.3%), Intermediate (73.0%), Unfavourable (10.0%)
Stone, 2017 [20]	Midostaurin (*N* = 360)	SOC therapy + placebo (*N* = 357)	Patients aged 18–59 years with newly diagnosed AML, with not previously antineoplastic therapy, with a bilirubin level of less than 2.5 times the upper limit of the normal range.	47.1 (range: 19.0–59.8.0.8)	48.6 (range: 18.0–60.9.0.9)	55.5%	De novo (100.0%)	ITD only (77.4), TKD only (22.6)	Favourable (5.3%), Intermediate (68.6%), Unfavourable (7.1%)
Wang, 2022 [21]	Gilteritinib + Azacitidine (*N* = 74)	SOC therapy + Azacitidine (*N* = 49)	Patients aged ≥ 18 years previously untreated for AML, positive for a FLT3 mutation (ITD and/or TKD), and ineligible for intensive induction chemotherapy. Patients with acute promyelocytic leukemia, BCR-ABL–positive leukemia, clinically active central nervous system leukemia, or major surgery/radiation therapy 4 weeks before the first study dose were excluded.	78.0 (range: 59–90)	76.0 (range 61–88)	43.1%	NA	ITD only (79.6), TKD only (17.1), ITD with TKD (3.3)	Favourable (1.6%), Intermediate (70.7%), Unfavourable (10.6%)
Wang, 2024 [22]	Gilteritinib (*N* = 116)	Investigator’s choice of salvage chemotherapy (*N* = 118)	Patients aged ≥ 18 years with primary AML or AML secondary to myelodysplastic syndromes who were refractory to or had relapsed after first-line AML therapy (≥ 1 cycle of standard dose anthracycline containing induction therapy or other induction therapy considered the optimum choice per investigator assessment) and positive for FLT3-ITD or FLT3-TKD D835 or I836 mutations in bone marrow or whole blood. Patients diagnosed with acute promyelocytic leukemia, BCR::ABL1-positive leukemia, AML secondary to prior chemotherapy, or active central nervous system disease were excluded.	51.5 (SD: ±16.1)	49.5 (SD: ±14.6)	53.8%	NA	ITD only (87.2), TKD only (9.0), ITD with TKD (3.8)	Favourable (10.3%), Intermediate (75.2%), Unfavourable (2.1%)

SOC: standard of care, ITD: internal tandem duplication, AML: acute myeloid leukemia, TKD: tyrosine kinase domain

### Outcomes

The safety analysis was performed by analyzing patients according to the actual treatment received, including the frequency and severity of treatment-emergent adverse events (TEAEs) across all studies. An adverse event (AE) was defined as any unintended medical occurrence in a participant who received the study drug or underwent study procedures, regardless of a causal relationship with the treatment. This included any unexpected sign (e.g., abnormal laboratory findings), symptom, or disease that occurred in temporal association with the investigational product, whether or not it was related to the treatment. A serious adverse event (SAE) was defined as any adverse event that resulted in death, was life-threatening, caused significant disability/incapacity, led to congenital anomalies or birth defects, required hospitalization or prolonged hospitalization, or was deemed medically important by the investigator or sponsor.

The safety/tolerability were analyzed in all patients who received ≥ 1 dose of the study treatment. The collection of adverse events generally began at the time of informed consent and continued through the 30-day follow-up visit [19]. All studies included in the review reported treatment-emergent adverse events (TEAEs), classifying them in at least two ways: by any grade and by severity, with a particular focus on events graded 3 or higher. The grading system followed the National Cancer Institute (NCI)-CTCAE guidelines, with grade 1 indicating mild events, grade 2 moderate events, grade 3 severe events, grade 4 life-threatening events, and grade 5 events resulting in death. All the studies reported grade ≥ 3 adverse events, with the exception of Stone et al., which did not provide safety data on adverse events of all grades [20].

Only one study provided a detailed classification of adverse events by grade, reporting them separately for grades 1–2, 3, 4, and 5 [16]. Several studies reported TEAEs distinguishing between all AEs and those of Grade 3 or higher [17–19, 22, 23]. Another trial reported only Grade 3, 4, or 5 AEs [20]. Additionally, only one study included a p-value, calculated using Fisher’s exact test [20] (Supplementary Table 1).

None of the included studies conducted a stratified analysis of TEAEs based on relevant demographic or clinical characteristics, such as age, sex, or comorbidities. As a result, it was not possible to assess how these factors might influence the frequency or severity of adverse events in different patient subgroups.

**Fig. 3:**
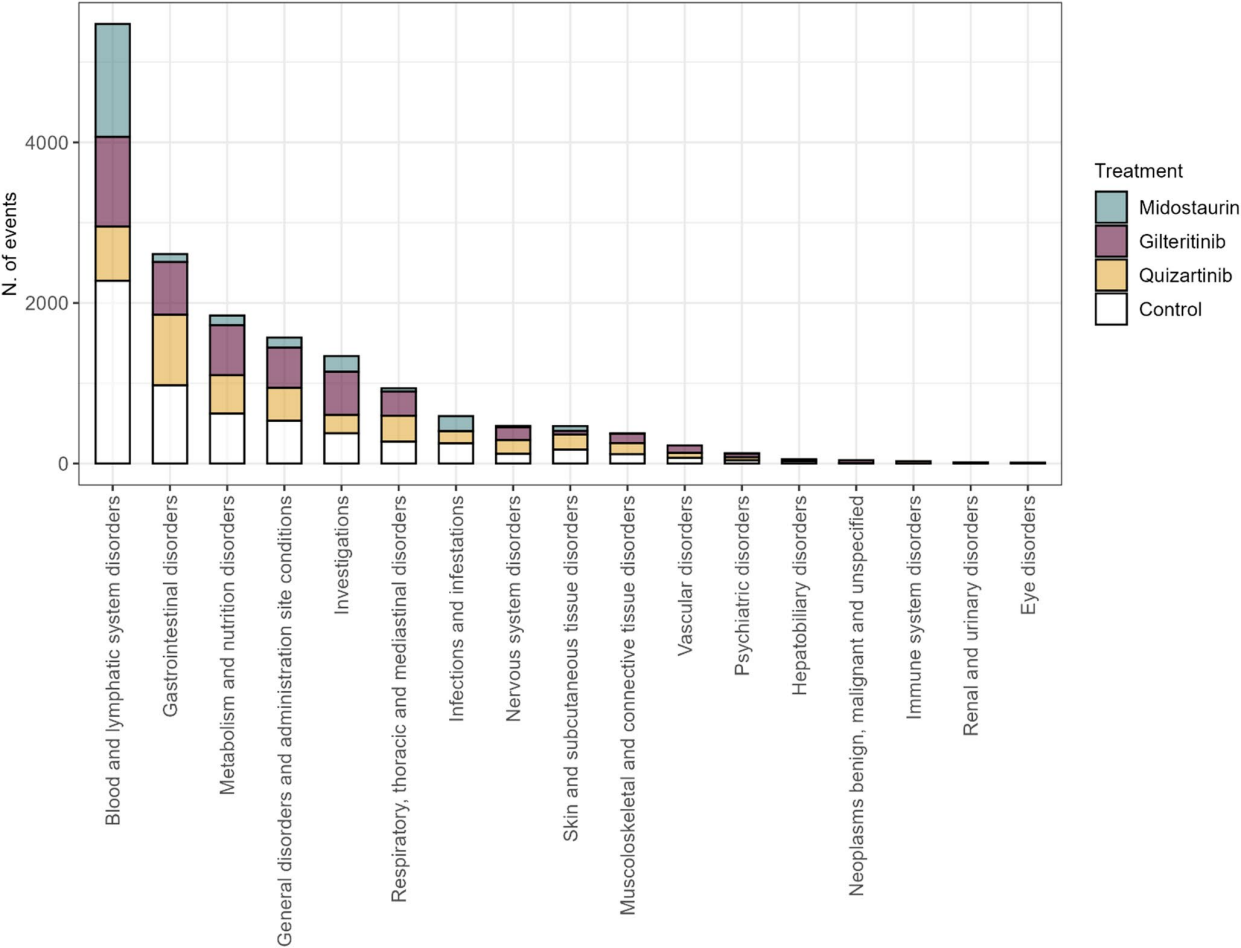
Distribution of adverse events of any grade reported in the studies included, categorized by System Organ Class (SOC) and stratified by treatment

Aggregating the safety data from the studies included in our systematic review, the most frequently reported adverse events of any grade were hematological adverse events, classified under the System Organ Class (SOC) Blood and lymphatic system disorders (*N* = 5474, 58.4% of them related to FLT3 inhibitors), followed by Gastrointestinal disorders (*N* = 2609, 62.7% of them related to FLT3 inhibitors), Metabolism and nutrition disorders (*N* = 1844, 66.2% of them related to FLT3 inhibitors), General disorders and administration site conditions (*N* = 1568, 66.1% of them related to FLT3 inhibitors), and Investigations (*N* = 1338, 71.9% of them related to FLT3 inhibitors) (Fig. 3).

The reported hematological adverse events included anemia, febrile neutropenia, leukocytosis, leukopenia, neutropenia, pancytopenia, and thrombocytopenia. Among the most frequently observed non-hematological adverse events were gastrointestinal disorders (such as abdominal pain, constipation, diarrhea, nausea, stomatitis, and vomiting), as well as fatigue, pyrexia, elevated ALT and AST levels, hypokalemia, myalgia, and headache (Table 2). Supplementary Tables 2 and Supplementary Table 3 provide details on the relative frequency of all recorded adverse events during the included trials, specifically those of grade ≥ 3 and those of any grade, respectively.

**Table 2:** Comparison of the most common adverse events across the five most frequently reported system organ classes (SOCs) between each treatment and control groups

Adverse event	Midostaurin *N* = 385 (100.0)	Gilteritinib *N* = 432 (100.0)	Quizartinib *N* = 513 (100.0)	Control *N* = 1037 (100.0)
*Blood and lymphatic system disorders*				
Thrombocytopenia	346 (89.9)	298 (69.0)	124 (24.2)	549 (52.9)
Neutropenia	341 (88.6)	210 (48.6)	162 (31.6)	509 (49.1)
Febrile neutropenia	290 (75.3)	210 (48.6)	198 (38.6)	511 (49.3)
Anemia	335 (87.0)	265 (61.3)	117 (22.8)	488 (47.1)
Leukopenia	93 (24.2)	133 (30.8)	47 (9.2)	211 (20.3)
*Gastrointestinal disorders*				
Diarrhea	63 (16.4)	173 (40.0)	168 (32.7)	259 (25.0)
Nausea	28 (7.3)	158 (36.6)	206 (40.2)	248 (23.9)
Vomiting	7 (1.8)	108 (25.0)	145 (28.3)	142 (13.7)
Constipation	0	117 (27.1)	103 (20.1)	117 (11.3)
Abdominal pain	0	53 (12.3)	129 (25.1)	95 (9.2)
*Metabolism and nutrition disorders*				
Hypokalemia	49 (12.7)	167 (38.7)	171 (33.3)	266 (25.7)
Hypocalcemia	24 (6.2)	83 (19.2)	55 (10.7)	88 (8.5)
Decreased appetite	0	79 (18.3)	95 (18.5)	63 (6.1)
Hyponatremia	31 (8.1)	79 (18.3)	22 (4.3)	53 (5.1)
Hypophosphatemia	19 (4.9)	46 (10.6)	51 (9.9)	68 (6.6)
Hypomagnesemia	0	51 (11.8)	67 (13.1)	42 (4.1)
Hyperglycemia	0	78 (18.1)	0	29 (2.8)
*General disorders and administration site conditions*				
Pyrexia	5 (1.3)	222 (51.4)	204 (39.8)	241 (23.2)
Fatigue	41 (10.6)	84 (19.4)	124 (24.2)	109 (10.5)
Oedema peripheral	9 (2.3)	88 (20.4)	81 (15.8)	85 (8.2)
Pain	47 (12.2)	0	0	44 (4.2)
Asthenia	0	65 (15.0)	0	17 (1.6)
*Investigations*				
Lymphopenia	136 (35.3)	22 (5.1)	0	180 (17.4)
ALT increased	52 (13.5)	148 (34.3)	74 (14.4)	100 (9.6)
AST increased	8 (2.1)	170 (39.4)	28 (5.5)	57 (5.5)

### Metanalysis

We pooled the results of clinically relevant adverse events belonging to the SOC Blood and lymphatic system disorders to compare the occurrence risk between treatment vs. control groups. All metanalyses were performed using the fixed-effect model, except for the one related to neutropenia, which, due to high heterogeneity (I^2^ = 63.4%), was conducted using a random-effects model.

**Fig. 4:**
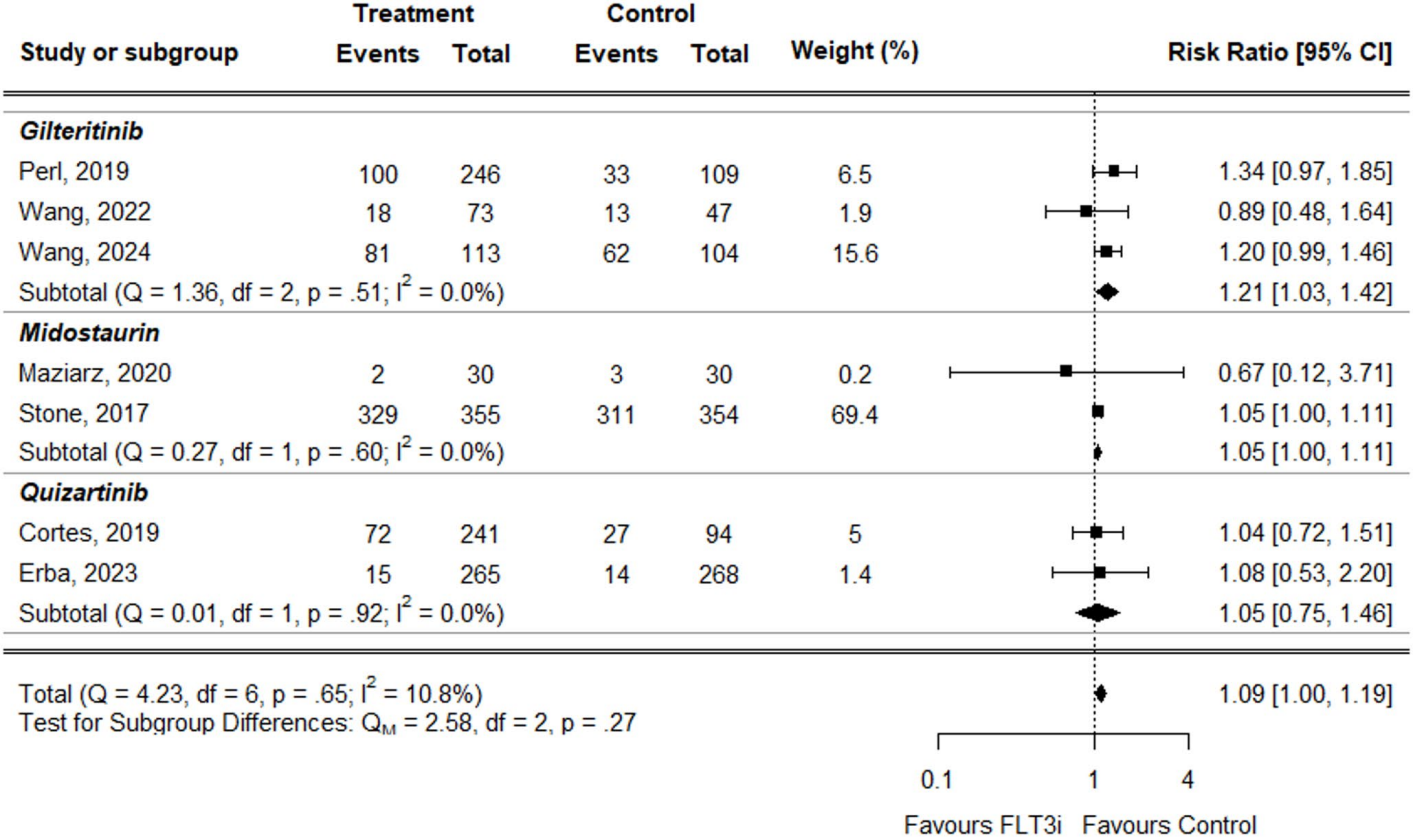
Forest plot for anemia data

For anemia, the overall Risk Ratio (RR) was slightly higher in treatment groups compared to controls, but the difference was not statistically significant (RR = 1.09, 95% CI: 1.00 to 1.19) (Fig. 4). This result was consistent across individual treatments: midostaurin (RR = 1.05, 95% CI: 1.00–1.11) and quizartinib (RR = 1.05, 95% CI: 0.75–1.46). Only gilteritinib showed a slightly statistically significant increased risk (RR = 1.21, 95% CI: 1.03–1.42).

**Fig. 5:**
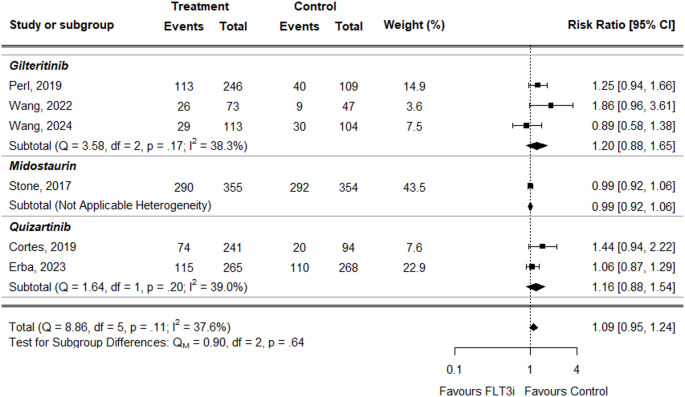
Forest plot of febrile neutropenia data

For febrile neutropenia, the overall RR was higher in the treatment groups but not statistically significant (RR = 1.09, 95% CI: 0.95–1.24) (Fig. 5). Individual treatments showed similar trends: gilteritinib (RR = 1.20, 95% CI: 0.88–1.65), midostaurin (RR = 0.99, 95% CI: 0.92–1.06), and quizartinib (RR = 1.16, 95% CI: 0.88–1.54).

**Fig. 6:**
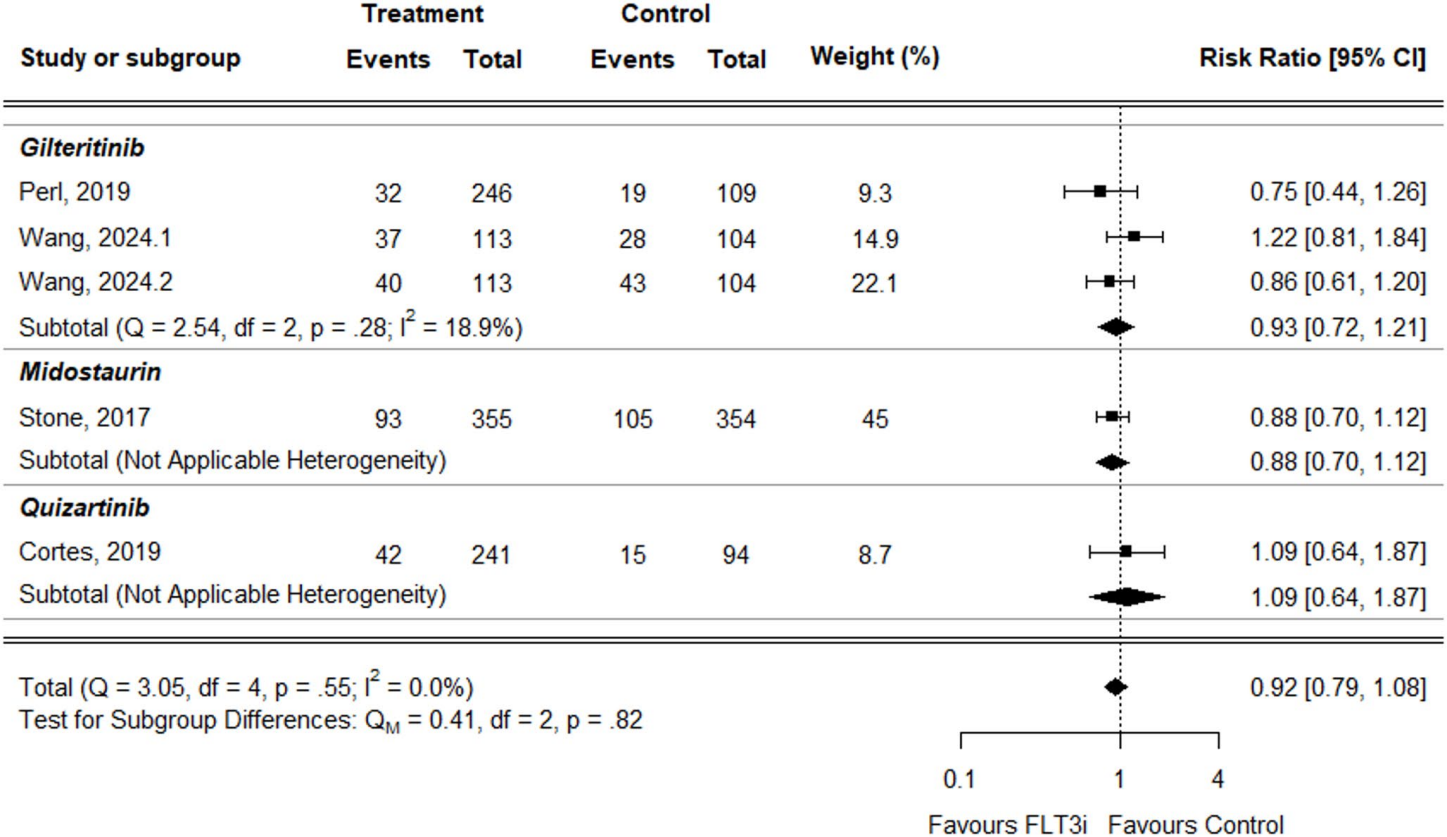
Forest plot of leukopenia data

For leukopenia, the overall RR was slightly lower in treatment groups than controls but not statistically significant (RR = 0.92, 95% CI: 0.79–1.08) (Fig. 6). This was consistent across treatments: gilteritinib (RR = 0.93, 95% CI: 0.72–1.21) and midostaurin (RR = 0.88, 95% CI: 0.70–1.12). Only quizartinib showed a slightly increased RR, but again not statistically significant (RR = 1.09, 95% CI: 0.64–1.87).

**Fig. 7:**
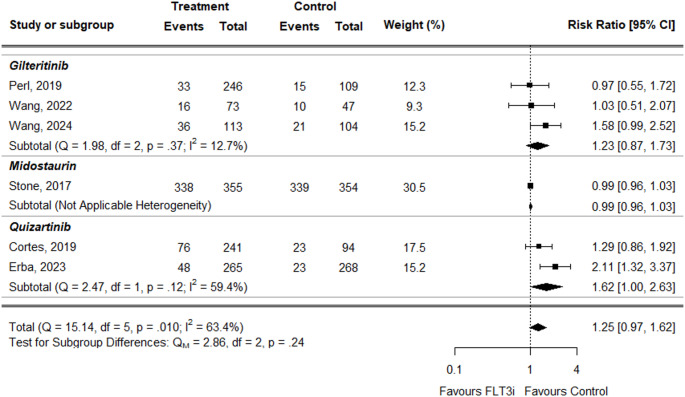
Forest plot of neutropenia data

For neutropenia, the overall RR was higher in treatment groups but not statistically significant (RR = 1.25, 95% CI: 0.97–1.62) (Fig. 7). This trend was reflected in the individual treatments: gilteritinib (RR = 1.23, 95% CI: 0.87–1.73) and quizartinib (RR = 1.62, 95% CI: 1.00–2.63). The only study evaluating midostaurin reported a slightly lower RR in treatment vs. control, but the result was not statistically significant (RR = 0.99, 95% CI: 0.96–1.03).

**Fig. 8:**
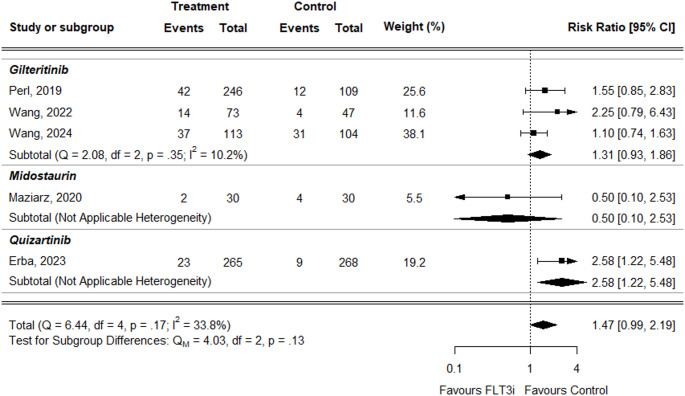
Forest plot of neutrophil count decreased data

For neutrophil count decreased, the overall RR was higher in treatment groups but did not reach statistical significance (RR = 1.47, 95% CI: 0.99–2.19) (Fig. 8). The result for gilteritinib was consistent (RR = 1.31, 95% CI: 0.93–1.96). For midostaurin, the RR was lower but not statistically significant (RR = 0.50, 95% CI: 0.10–2.53). In contrast, for quizartinib, only the study by Erba et al. was available, which showed a statistically significant increased risk in the treatment group (RR = 2.58, 95% CI: 1.22–5.48).

Notably, neutropenia and decreased neutrophil count, despite being related outcomes, are presented separately (as in studies such as Perl 2019, Wang 2022, Wang 2024, and Erba 2023) to differentiate between the more severe condition (i.e., neutropenia) and the potentially less clinically impactful decreased neutrophil count.

**Fig. 9:**
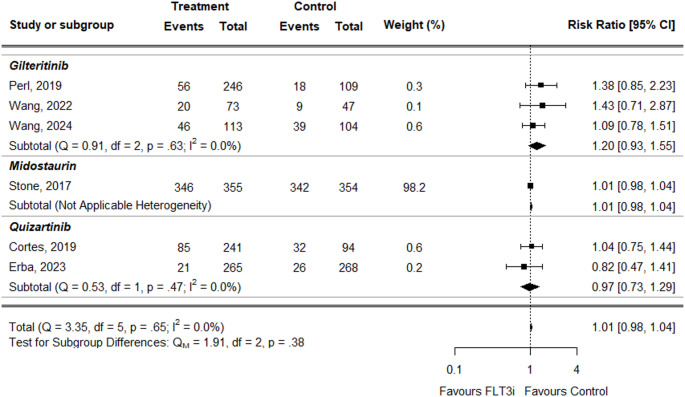
Forest plot of thrombocytopenia data

For thrombocytopenia, the overall RR was similar between treatment and control groups and not statistically significant (RR = 1.01, 95% CI: 0.98–1.04) (Fig. 9). Individual treatment RRs were: gilteritinib (RR = 1.20, 95% CI: 0.93–1.55), midostaurin (RR = 1.01, 95% CI: 0.98–1.04), and quizartinib (RR = 0.97, 95% CI: 0.73–1.29), with none showing statistical significance.

Additionally, we pooled the results of clinically relevant adverse events belonging to the SOC Gastrointestinal disorders, General disorders and administration site conditions, Metabolism and nutrition disorders and Investigations to compare the occurrence risk between treatments vs controls (supplementary materials). We also grouped selected adverse events such as hypertension, infection, pneumonia, and sepsis or septic shock under the category “Other adverse events”.

Across these SOCs, the overall RR did not show a statistical significance. Also the results of RR for single treatments were not statistically significant. The only exception was the SOC Investigations, where the overall RR was significantly higher in treatment groups (RR = 1.48, 95% CI: 1.06–2.08). Additionally, ALT increased showed a significantly higher RR in treatment groups (RR = 2.40, 95% CI: 1.16–4.95) (Table 3 and Supplementary Figs. 3–7).

**Table 3:** Summary of pooled risk ratios for the most common adverse events across treatments

Adverse event	Risk Ratio (95% Confidence Interval)		
	Gilteritinib	Midostaurin	Quizartinib
**Gastrointestinal disorders**			
Diarrhea	1.53 (0.61–3.83)	1.01 (0.72–1.41)	0.85 (0.41–1.80)
Nausea	1.49 (0.25–8.79)	0.92 (0.22–3.85)	1.09 (0.36–3.29)
Stomatitis	0.66 (0.19–2.31)	-	0.95 (0.32–2.85)
**Metabolism disorders**			
Decreased appetite	0.68 (0.18–2.51)	-	2.83 (1.09–7.39)
Hyperglycemia	1.00 (0.48–2.06)	-	0.65 (0.16–2.67)
Hypocalcemia	1.69 (0.23–12.33)	1.14 (0.65–2.01)	0.23 (0.06–0.85)
Hypokalemia	1.04 (0.68–1.59)	0.81 (0.58–1.15)	1.19 (0.85–1.65)
Hypomagnesemia	2.23 (0.11–46.00)	-	0.74 (0.08–7.06)
Hyponatremia	2.13 (1.00–4.51)	1.34 (0.80–2.26)	6.67 (0.39–114.48)
Hypophosphatemia	2.22 (0.78–6.33)	0.65 (0.37–1.14)	1.05 (0.61–1.82)
**General disorders**			
Asthenia	1.97 (0.49–7.99)	-	-
Fatigue	1.33 (0.27–6.48)	0.85 (0.54–1.32)	5.77 (1.06–31.36)
Oedema peripheral	1.00 (0.08–11.84)	1.00 (0.02–48.82)	0.77 (0.10–5.72)
Pain	0.89 (0.08–9.67)	1.07 (0.73–1.56)	
Pyrexia	1.64 (0.63–4.22)	3.00 (0.13–70.83)	0.82 (0.43–1.58)
**Investigations**			
ALT increased	2.40 (1.16–4.95)	1.36 (0.90–2.04)	1.06 (0.54–2.10)
AST increased	3.19 (0.74–13.75)	2.00 (0.40–10.11)	2.36 (0.62–9.03)
Electrocardiogram QT prolonged	-	-	3.29 (1.00–10.85)
Lymphopenia	0.79 (0.46–1.38)	0.87 (0.65–1.16)	-
**Other adverse events**			
Hypertension	2.22 (0.78–6.33)	-	0.73 (0.37–1.46)
Infection	-	1.04 (0.90–1.20)	0.62 (0.21–1.86)
Pneumonia	1.44 (0.96–2.15)	0.96 (0.59–1.58)	1.11 (0.75–1.67)
Sepsis or septic shock	0.52 (0.15–1.82)	-	0.71 (0.34–1.49)

## Discussion

The aim of this systematic review and meta-analysis was to evaluate the clinical safety profile of FLT3 inhibitors in patients with any subtype of acute myeloid leukemia (AML). Specifically, we aimed to identify and analyze adverse events reported in clinical trials, with the goal of comparing FLT3 inhibitors both to control treatments and among themselves. Seven trials were identified from the review search. Two studies investigated midostaurin, a first-generation FLT3 inhibitor, while five studies evaluated second-generation inhibitors, including gilteritinib in three trials and quizartinib in two. The primary distinction between first- and next-generation FLT3 inhibitors lies in their potency and selectivity for FLT3 and its downstream signaling pathways. First-generation inhibitors (i.e., sunitinib, sorafenib, and midostaurin) exhibit broader kinase activity, targeting not only FLT3 but also other receptors including KIT, PDGFR, VEGFR, RAS/RAF, and JAK2. This off-target activity suggests a potentially higher toxicity profile in patients with FLT3-mutated AML [24]. The second generation inhibitors (i.e., quizartinib, crenolanib, and gilteritinib) are more selective and potent, and are therefore potentially associated with fewer off-target toxicities.

Three trials included in our review focused exclusively on patients with FLT3 internal tandem duplication (FLT3-ITD) mutations and four trials included patients with either FLT3-ITD and/or FLT3 tyrosine kinase domain (TKD) point mutations. While the prognostic significance of FLT3-TKD mutations in AML remains unclear, the FLT3-ITD mutation is well recognized as being associated with poor outcomes, particularly with regard to relapse risk and overall survival [25–28]. These considerations should be taken into account, as the study populations—both within individual trials and in our overall analysis—are not entirely homogeneous. Such differences may have influenced the observed results.

Female patients represented the majority in 5 out of the 7 included trials. In line with our aims, it would be valuable to stratify the results by sex, given the known sex-related differences across various aspects of AML. In fact, numerous studies suggest that sex differences influence both efficacy and safety outcomes in patients with AML. Firstly, significant sex differences have been observed in the genomic aberrations underlying AML pathogenesis, which may be partly attributed to differences in environmental exposures and hormonal influences, particularly estrogen and androgen signaling [29, 30]. The sex differences also manifest in prognosis and survival rate differences between female and male AML patients [31, 32]. Some studies have investigated sex-related differences in response to treatment with FLT3 inhibitors, but these analyses have been limited to efficacy outcomes (i.e., overall survival and/or event-free survival), with no available data on sex-specific safety profiles. For instance, the RATIFY trial, which evaluated the addition of midostaurin to standard chemotherapy in FLT3-mutated patients, showed a significant overall survival (OS) benefit in males, while no benefit was observed in females [20]. Similarly, the AGILE trial reported improved event-free survival (EFS) and OS for males treated with ivosidenib and azacitidine, but not for females [33]. Conversely, data from the QUANTUM-First and ADMIRAL trials suggest a possible sex-based reversal: quizartinib and gilteritinib were associated with significant OS benefits in females, but not in males [17, 19]. These findings underscore the importance of further exploring sex-specific differences not only in efficacy but also in the safety profiles of FLT3 inhibitors, an area that remains largely unexamined. As previously suggested, future research should aim to design clinical trials that specifically address sex differences in AML. These studies should not only stratify patients by sex but also be adequately powered through larger sample sizes to investigate the mechanisms underlying observed differences [34].

The age of patients included in the studies varied considerably, with median ages ranging from 48 to 78 years across trials. It is also important to note all the studies focused exclusively on adult patients, although the definition of adulthood differed among trials. For example, Erba et al. defined adult patients as those aged 18 to 75 years, Maziarz et al. used a range of 18 to 70 years, while Perl et al., E. Wang et al., and J. Wang et al. included all patients aged 18 and above. These latter studies therefore also included elderly patients. While a worse prognosis and reduced treatment response are generally expected in older individuals, available data remain limited, both due to small patient numbers and underpowered analyses in some of the studies with negative results [35]. It would be useful for future clinical trials and observational studies to provide specific data on elderly patients, in order to better understand treatment outcomes and safety profiles of FLT3 inhibitors in this growing and often underrepresented population.

The safety profile observed across the included studies reveals a consistent pattern of treatment-emergent adverse events (TEAEs), with hematological and gastrointestinal disorders (e.g., nausea, diarrhea) which are the most frequently reported. This trend aligns with the known safety profiles of FLT3 inhibitors. Notably, the incidence of hematological adverse events may also be influenced by the underlying disease progression, making it challenging to distinguish between treatment-related effects and the natural course of the AML. Moreover, the potential impact of resistance mechanisms to FLT3 inhibitors should also be taken into account. These mechanisms are generally categorized as either primary or secondary resistance [36, 37]. Primary resistance may result from differential inhibitor potency against various types of FLT3 mutations, particularly between FLT3-ITD and FLT3-TKD mutations. In contrast, secondary resistance mechanisms include FLT3 ligand (FL)-mediated resistance, the emergence of resistance-associated mutations, and overexpression of the FLT3 receptor itself [38].

Importantly, none of the included studies conducted stratified analyses of adverse events based on key demographic or clinical variables such as age, sex, or comorbidities. This represents a critical gap, as such factors could significantly influence both the incidence and severity of TEAEs.

The pooled analysis of adverse events within the Blood and Lymphatic System Disorders SOC revealed a consistent pattern across treatments, although most findings did not reach statistical significance. Among individual FLT3 inhibitors, only gilteritinib demonstrated a statistically significant increase in anemia risk (RR = 1.21, 95% CI 1.03–1.42), suggesting a potential drug-specific effect that warrants further investigation. None of the three FLT3 inhibitors showed a clear association with febrile neutropenia, that remains a critical complication in AML treatment. These findings are clinically relevant, as febrile neutropenia is a relatively common but serious complication of treatment and post-treatment adverse events for hematologic cancer patients, even in the absence of statistically significant differences [39]. No statistically significant differences were found in overall risk ratios between treatment and control groups for leukopenia, neutropenia, neutrophil count decreased and thrombocytopenia. One possible explanation is that these adverse events may be largely attributable to the underlying disease process or concomitant chemotherapy, rather than to the FLT3 inhibitor itself. Moreover, the heterogeneity in reporting practices, treatment regimens, and patient characteristics across studies may have limited the ability to detect significant differences [40, 41].

Beyond hematologic adverse events, we also evaluated adverse events within other SOCs, including Gastrointestinal disorders, General disorders and administration site conditions, Metabolism and nutrition disorders, and Investigations. In general, no statistically significant differences were found in overall risk ratios between treatment and control groups and between each treatment. However, the SOC Investigations showed a statistically significant increased risk in treatment arms. Notably, elevated ALT levels were significantly more frequent among patients treated with gilteritinib (RR = 2.40, 95% CI 1.16–4.95), indicating a potential risk of hepatotoxicity associated with the drug. Both in vitro and in vivo studies – aligning with clinical findings – have demonstrated that gilteritinib can induce liver injury. Mechanistically, gilteritinib appears to promote hepatocyte apoptosis through activation of the p53-dependent mitochondrial damage pathway. Further investigations have shown that this process involves an upregulation of the DDIT4 protein in a p53-dependent manner. The accumulation of DDIT4 in liver cells contributes significantly to gilteritinib-induced hepatotoxicity, suggesting that targeting DDIT4 may offer a promising strategy for mitigating this adverse event [42].

A selection of clinically important adverse events grouped under “Other adverse events” such as hypertension, infections, pneumonia, and sepsis or septic shock, were also evaluated. No significant differences were observed, although the clinical relevance of these complications remains high in vulnerable AML populations, even in the absence of statistical significance [43, 44].

## Strengths and limitations

This systematic review and metanalysis provides a comprehensive overview of the safety profile of FLT3 inhibitors in adult patients with acute myeloid leukemia. One of the key strengths of this study is its ability to aggregate data from multiple randomized controlled trials to offer a clearer picture of the adverse events associated with these therapies.

However, the study highlights a notable gap in the reporting of safety data, which limits our understanding of their complete safety profile. In fact, a notable limitation across the studies was the inconsistency in how adverse events were reported. While all studies assessed TEAEs, only one provided a detailed safety data by grade (1 through 5), and some studies reported only grade ≥ 3 events. This heterogeneity complicates the interpretation and comparison of safety data and may result in an underestimation of low-grade but clinically relevant adverse events that affect quality of life. Moreover, all the included studies did not provide patient-level safety data, which prevented us from performing subgroup analyses to investigate the impact of variables such as age, sex, disease stage, and other confounders on adverse event occurrence. Importantly, safety data from combination regimens should be interpreted with caution, as adverse events cannot be reliably attributed to the FLT3 inhibitor alone.

Finally, due to the relatively short follow-up periods of the included trials, rare and long-term adverse events may not have been detected. This highlights the need for longer-term observational studies to capture such events and provide a more complete safety profile for FLT3 inhibitors. The limited scope of clinical trials in detecting infrequent but potentially serious adverse events calls for further research to complement these findings and enhance patient safety monitoring in the long term.

## Conclusion

Our findings indicate that FLT3 inhibitors are not associated with a statistically significant increase in the risk of adverse events compared to standard treatments. Furthermore, no meaningful differences in adverse event risk were observed among the three FLT3 inhibitors evaluated – midostaurin (first-generation), gilteritinib, and quizartinib (both second-generation). The only exception was a significantly higher risk of hepatotoxicity associated with gilteritinib, as reflected by increased ALT levels in treated patients.

Specifically to the hematologic toxicity, it is important to consider that the background risk of hematologic toxicity in AML is inherently high and can be confounded by disease progression, making it difficult to attribute causality solely to the treatment. Furthermore, the variability in adverse event reporting and the limited statistical power of subgroup analyses limit the generalizability of our findings.

In our opinion, future studies should aim to stratify safety outcomes based on demographic and clinical characteristics, including age, performance status, and mutation type (e.g., FLT3-ITD vs. TKD), to better understand differential safety patterns. Moreover, incorporating real-world data and long-term follow-up will be critical for a more comprehensive assessment of safety in clinical practice.

## Supplementary Information

Below is the link to the electronic supplementary material.

Supplementary Material 1

## Data Availability

No datasets were generated or analysed during the current study.
