# Supplementary material for: Safety profile of FLT3 inhibitors in acute myeloid leukemia: a systematic review and meta-analysis of adverse events
Source: Clin Exp Med. 2026 Feb 23;26(1):167. doi: 10.1007/s10238-026-02093-8 (PMC12953341; doi:10.1007/s10238-026-02093-8)
Supplement: Supplementary file 1 — Supplementary Material 1 [file 10238_2026_2093_MOESM1_ESM.docx]

Supplementary Textbox 1. MEDLINE (PubMed) search strategy for FLT3 inhibitors in patients with AML.

| **Query** |
| --- |
| #5 AND #11 AND #12 |
| (((((randomized controlled trial[Publication Type]) OR (controlled clinical trial[Publication Type])) OR (randomized[Title/Abstract])) OR (randomly[Title/Abstract])) OR (trial[Title/Abstract])) OR (groups[Title/Abstract]) |
| #6 OR #7 OR #8 OR #9 OR #10 |
| ((gilteritinib[Title/Abstract]) OR (ASP2215[Title/Abstract])) OR (ASP-2215[Title/Abstract]) |
| ((quizartinib[Title/Abstract]) OR (AC-220[Title/Abstract])) OR (AC220[Title/Abstract]) |
| ((midostaurin[Title/Abstract]) OR (PKC-412[Title/Abstract])) OR (PKC412[Title/Abstract]) |
| ((FLT3[Title/Abstract]) OR (FLT-3[Title/Abstract])) OR (midostaurin[Title/Abstract]) |
| fms like tyrosine kinase 3[MeSH Terms] |
| #1 OR #4 |
| #2 AND #3 |
| ("myelo*"[Text Word] OR "nonlympho*"[Text Word] OR "granulocytic*"[Text Word] OR "monocyt*"[Text Word] OR "megakaryoblast*"[Text Word] OR "promyelocyt*"[Text Word] OR "erythroblast*"[Text Word]) AND ("leuk*em*"[Text Word] OR "leuc*"[All Fields]) |
| acut*[Text Word] |
| acute myeloid leukemia[MeSH Terms] |

Supplementary Textbox 2. MEDLINE (PubMed) search strategy for FLT3 inhibitors in patients with AML.

('acute myeloid leukemia'/exp OR ('acut*':ti,ab,kw,de,dn,df,mn,tn AND ('myelo*':ti,ab,kw,de,dn,df,mn,tn OR 'nonlympho*':ti,ab,kw,de,dn,df,mn,tn OR 'granulocytic*':ti,ab,kw,de,dn,df,mn,tn OR 'monocyt*':ti,ab,kw,de,dn,df,mn,tn OR 'megakaryoblast*':ti,ab,kw,de,dn,df,mn,tn OR 'promyelocyt*':ti,ab,kw,de,dn,df,mn,tn OR 'erythroblast*':ti,ab,kw,de,dn,df,mn,tn) AND ('leuk*em*':ti,ab,kw,de,dn,df,mn,tn OR 'leuc*'))) AND ('cd135 antigen'/exp OR 'flt3':ti,ab,kw OR 'flt-3':ti,ab,kw OR 'midostaurin':ti,ab,kw OR 'pkc-412':ti,ab,kw OR 'pkc412':ti,ab,kw OR 'quizartinib':ti,ab,kw OR 'ac-220':ti,ab,kw OR 'ac220':ti,ab,kw OR 'gilteritinib':ti,ab,kw OR 'asp2215':ti,ab,kw OR 'asp-2215':ti,ab,kw) AND ('randomized controlled trial':it OR 'controlled clinical trial':it OR 'randomized':ti,ab,kw OR 'randomly':ti,ab,kw OR 'trial':ti,ab,kw OR 'groups':ti,ab,kw)

Supplementary Textbox 3. Cochrane search strategy for FLT3 inhibitors in patients with AML.

(acut* AND (myelo* OR nonlympho* OR granulocytic* OR monocyt* OR megakaryoblast* OR promyelocyt* OR erythroblast*) AND (leukem* OR leuc*)) AND (FLT3 OR "FLT-3" OR midostaurin OR PKC-412 OR PKC412 OR quizartinib OR AC-220 OR AC220 OR gilteritinib OR ASP2215 OR ASP-2215) AND (randomized OR trial OR "randomly allocated" OR "controlled clinical trial" OR "clinical trial")


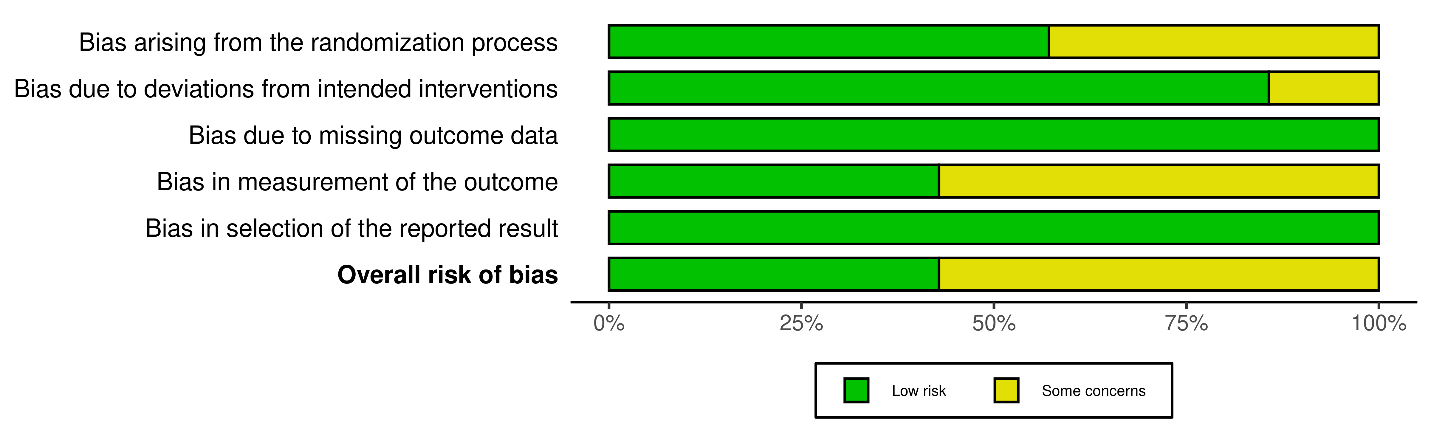


Supplementary Figure 1. Risk of bias graph of included trials.


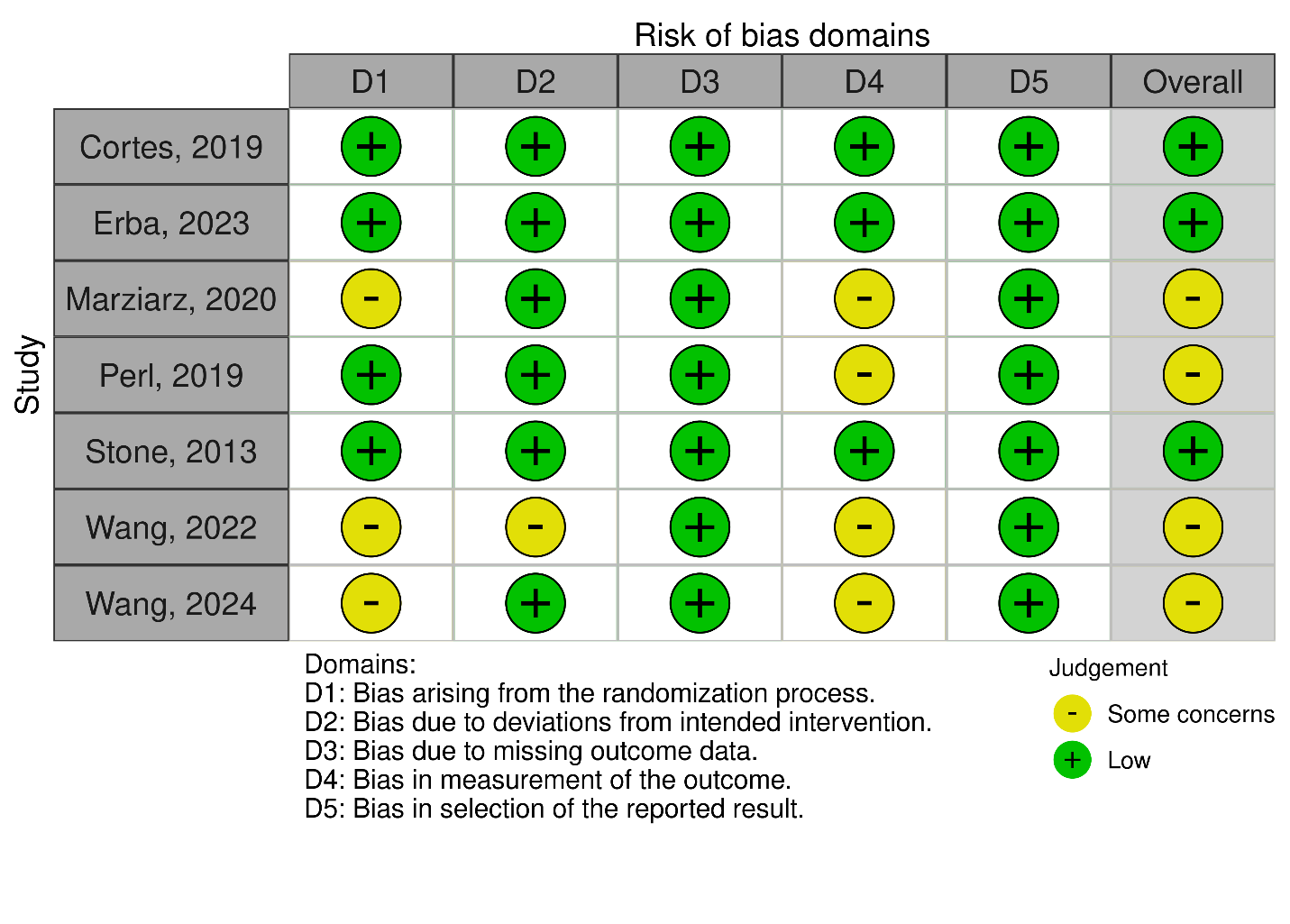


**Supplementary Figure 2.** Risk of bias summary of included trials.

**Supplementary Table 1.** Data sources and methods of calculation for the safety outcome. Filled circle indicates that the study explicitly reported data for the specific safety outcome in the corresponding column; the “x” indicates that the study did not report data for the specific safety outcome; “Cal” indicates that the safety outcome was not explicitly reported but could be calculated from the data provided in the study; “NA” is not applicable.

| **Author, year** | **Grade 1-2** | **Grade 3** | **Grade 4** | **Grade 5** | **All grades** | **≥3 grade** | **p-value** |
| --- | --- | --- | --- | --- | --- | --- | --- |
| Cortes, 2019 | ● | ● | ● | ● | Cal | Cal | x |
| Erba, 2023 | x | x | x | x | ● | ● | x |
| Maziarz, 2020 | x | x | x | x | ● | ● | x |
| Perl, 2019 | x | x | x | x | ● | ● | x |
| Stone, 2017 | x | x | x | x | x | ● | ● |
| Wang, 2022 | x | x | x | x | ● | ● | x |
| Wang, 2024 | x | x | x | x | ● | ● | x |

**Supplementary Table 2.** Percentage of grade 3 and above adverse events, classified by System Organ Class and stratified by each study included in the review. Note: Data from Stone et al. include only grade 3 and 4 adverse events, excluding grade 5.

| **Adverse events**  **(≥3 grade)** | **Cortes, 2019** | | **Erba, 2023** | | **Maziarz, 2020** | | **Perl, 2019** | | **Stone, 2017*** | | **Wang, 2022** | | **Wang, 2024** | |
| --- | --- | --- | --- | --- | --- | --- | --- | --- | --- | --- | --- | --- | --- | --- |
|  | **Quizartinib (N=245)** | **Control (N=122)** | **Quizartinib (N=268)** | **Control (N=271)** | **Midostaurin (N=30)** | **Control (N=30)** | **Gilteritinib (N=246)** | **Control (N=109)** | **Midostaurin (N=355)** | **Control (N=354)** | **Gilteritinib (N=73)** | **Control (N=47)** | **Gilteritinib (N=113)** | **Control (N=104)** |
| **Blood and lymphatic system disorders** |  |  |  |  |  |  |  |  |  |  |  |  |  |  |
| Anemia | 29.9% | 28.7% | 5.7% | 5.2% | 6.7% | 10.0% | 40.7% | 30.3% | 92.7% | 87.9% | 24.7% | 27.7% | 71.7% | 59.6% |
| Bone marrow event |  |  |  |  |  |  |  |  | 0.3% | 1.4% |  |  |  |  |
| Febrile neutropenia | 30.7% | 21.3% | 43.4% | 41.0% |  |  | 45.9% | 36.7% | 81.7% | 82.5% | 35.6% | 19.1% | 25.7% | 28.8% |
| Leukocytosis | 2.9% | 3.2% |  |  |  |  |  |  |  |  |  |  |  |  |
| Leukopenia | 17.4% | 16.0% |  |  |  |  | 13.0% | 17.4% | 26.2% | 29.7% |  |  | 68.1% | 68.3% |
| Neutropenia | 31.5% | 24.5% | 26.8% | 11.9% | 6.7% | 13.3% | 30.5% | 24.8% | 95.2% | 95.8% | 41.1% | 29.8% | 64.6% | 50.0% |
| Pancytopenia | 4.6% | 0.0% |  |  |  |  |  |  |  |  |  |  |  |  |
| Thrombocytopenia | 35.3% | 34.0% | 7.9% | 9.7% |  |  | 44.7% | 41.3% | 97.5% | 96.6% | 45.2% | 38.3% | 78.8% | 76.0% |
| **Gastrointestinal disorders** |  |  |  |  |  |  |  |  |  |  |  |  |  |  |
| Abdominal pain | 2.1% | 1.1% | 2.3% | 1.9% |  |  | 2.0% | 0.0% |  |  |  |  |  |  |
| Constipation | 0.0% | 0.0% | 0.4% | 0.0% |  |  | 0.8% | 0.0% |  |  | 0.0% | 0.0% |  |  |
| Diarrhea | 1.7% | 3.2% | 3.8% | 3.7% | 3.3% | 10.0% | 3.7% | 2.8% | 15.8% | 15.3% | 6.8% | 0.0% | 3.5% | 2.9% |
| Dyspepsia |  |  | 0.4% | 0.7% |  |  |  |  |  |  |  |  |  |  |
| Nausea | 2.5% | 1.1% | 1.5% | 1.9% | 10.0% | 3.3% | 2.0% | 0.0% | 5.6% | 9.6% | 1.4% | 2.1% | 0.0% | 0.0% |
| Stomatitis | 2.1% | 4.3% | 4.5% | 3.0% |  |  | 2.4% | 3.7% |  |  |  |  |  |  |
| Vomiting | 3.3% | 1.1% | 0.0% | 1.5% | 3.3% | 6.7% | 0.4% | 0.0% |  |  | 2.7% | 0.0% | 0.9% | 1.0% |
| **General disorders** |  |  |  |  |  |  |  |  |  |  |  |  |  |  |
| Asthenia |  |  |  |  |  |  | 2.4% | 1.8% |  |  | 6.8% | 0.0% |  |  |
| Fatigue | 7.9% | 1.1% | 0.4% | 0.0% | 0.0% | 3.3% | 2.4% | 1.8% | 9.0% | 10.5% |  |  |  |  |
| Mucositis/stomatitis |  |  |  |  |  |  |  |  | 6.2% | 7.9% |  |  |  |  |
| Oedema peripheral | 1.2% | 0.0% | 0.4% | 1.1% | 0.0% | 0.0% | 0.4% | 0.0% |  |  | 0.0% | 0.0% |  |  |
| Pain |  |  |  |  |  |  |  |  | 13.2% | 12.4% |  |  |  |  |
| Pain in extremity |  |  |  |  |  |  | 0.8% | 0.9% |  |  |  |  |  |  |
| Pyrexia | 2.5% | 4.3% | 4.5% | 4.9% | 3.3% | 0.0% | 3.3% | 3.7% |  |  | 9.6% | 0.0% | 6.2% | 2.9% |
| **Hepatobiliary disorders** |  |  |  |  |  |  |  |  |  |  |  |  |  |  |
| Hyperbilirubinemia |  |  |  |  |  |  |  |  | 7.0% | 7.9% |  |  |  |  |
| **Immune system disorders** |  |  |  |  |  |  |  |  |  |  |  |  |  |  |
| Graft-versus-host disease | 4.6% | 0.0% |  |  |  |  |  |  |  |  |  |  |  |  |
| **Infections and infestations** |  |  |  |  |  |  |  |  |  |  |  |  |  |  |
| Cellulitis | 3.3% | 0.0% |  |  |  |  |  |  |  |  |  |  |  |  |
| Clostridioides difficile  infection | 2.1% | 0.0% |  |  |  |  |  |  |  |  |  |  |  |  |
| Device-related infection | 3.3% | 5.3% |  |  |  |  |  |  |  |  |  |  |  |  |
| Infection |  |  |  |  |  |  |  |  | 52.4% | 50.3% |  |  |  |  |
| Sepsis or septic shock | 19.1% | 19.1% | 4.2% | 9.0% |  |  |  |  |  |  | 5.5% | 10.6% |  |  |
| Upper respiratory tract infection | 2.1% | 0.0% |  |  |  | 0.0% |  |  |  |  |  |  |  |  |
| Urinary tract infection | 4.1% | 0.0% |  |  |  |  |  |  |  |  |  |  |  |  |
| **Investigations** |  |  |  |  |  |  |  |  |  |  |  |  |  |  |
| ALT increased | 3.7% | 2.1% | 4.5% | 4.9% | 13.3% | 10.0% | 13.8% | 4.6% | 12.7% | 9.3% |  |  | 6.2% | 3.8% |
| AST increased |  |  | 2.6% | 1.1% | 13.3% | 6.7% | 14.6% | 1.8% |  |  | 5.5% | 0.0% | 5.3% | 4.8% |
| Blood alkaline  phosphatase increased |  |  |  |  |  |  | 2.8% | 0.0% |  |  |  |  |  |  |
| Blood creatine  phosphokinase increased |  |  |  |  |  |  | 6.5% | 0.0% |  |  |  |  |  |  |
| Blood lactate dehydrogenase increased |  |  |  |  |  |  |  |  |  |  |  |  | 4.4% | 2.9% |
| Electrocardiogram  QT prolonged | 4.1% | 0.0% | 3.0% | 1.1% |  |  |  |  |  |  |  |  |  |  |
| Lymphopenia |  |  |  |  |  |  |  |  | 38.4% | 44.0% |  |  | 16.8% | 21.2% |
| Weight decreased | 0.4% | 0.0% |  |  |  |  |  |  |  |  |  |  |  |  |
| Weight increased |  |  |  |  |  |  |  |  |  |  |  |  | 4.4% | 0.0% |
| **Metabolism and nutrition disorders** |  |  |  |  |  |  |  |  |  |  |  |  |  |  |
| Decreased appetite | 2.5% | 0.0% | 4.9% | 1.9% |  |  | 2.0% | 4.6% |  |  | 4.1% | 2.1% |  |  |
| Hyperglycaemia | 2.1% | 3.2% |  |  |  |  | 7.3% | 8.3% |  |  |  |  | 2.7% | 1.0% |
| Hypoalbuminemia |  |  |  |  |  |  | 1.2% | 1.8% |  |  |  |  |  |  |
| Hypocalcemia | 0.4% | 2.1% | 0.8% | 3.0% |  |  | 4.9% | 0.9% | 6.8% | 5.9% |  |  | 2.7% | 3.8% |
| Hypokalemia | 11.6% | 8.5% | 18.9% | 16.4% |  |  | 13.0% | 11.0% | 13.8% | 16.9% | 8.2% | 8.5% | 13.3% | 14.4% |
| Hypomagnesemia | 0.0% | 0.0% | 0.4% | 0.4% |  |  | 0.8% | 0.0% |  |  |  |  |  |  |
| Hyponatremia | 3.3% | 0.0% |  |  |  |  | 6.5% | 2.8% | 8.7% | 6.5% | 12.3% | 2.1% | 7.1% | 4.8% |
| Hypophosphatemia | 4.6% | 5.3% | 6.8% | 6.0% |  |  | 8.1% | 3.7% | 5.4% | 8.2% |  |  |  |  |
| **Muscoloskeletal disorders** |  |  |  |  |  |  |  |  |  |  |  |  |  |  |
| Arthralgia |  |  | 0.4% | 0.7% | 3.3% | 0.0% | 1.6% | 0.9% |  |  |  |  |  |  |
| Back pain |  |  | 0.0% | 0.7% |  |  | 0.8% | 0.9% |  |  |  |  |  |  |
| Myalgia | 4.1% | 4.3% |  |  |  |  | 0.4% | 0.0% |  |  |  |  |  |  |
| **Neoplasms** |  |  |  |  |  |  |  |  |  |  |  |  |  |  |
| Acute myeloid leukemia |  |  |  |  |  |  | 13.4% | 3.7% |  |  |  |  |  |  |
| **Nervous system disorders** |  |  |  |  |  |  |  |  |  |  |  |  |  |  |
| Dizziness | 0.0% | 0.0% |  |  | 0.0% | 0.0% | 0.4% | 0.0% |  |  |  |  |  |  |
| Dysgeusia |  |  |  |  |  |  | 0.0% | 0.0% |  |  |  |  |  |  |
| Headache | 0.4% | 0.0% | 0.0% | 0.7% | 0.0% | 0.0% | 1.2% | 0.0% |  |  |  |  |  |  |
| Syncope | 3.7% | 0.0% |  |  |  |  |  |  |  |  |  |  |  |  |
| Tremor |  |  |  |  | 0.0% | 0.0% |  |  |  |  |  |  |  |  |
| **Psychiatric disorders** |  |  |  |  |  |  |  |  |  |  |  |  |  |  |
| Insomnia |  |  | 0.0% | 0.0% | 0.0% | 0.0% | 0.0% | 0.0% |  |  |  |  |  |  |
| **Renal and urinary disorders** |  |  |  |  |  |  |  |  |  |  |  |  |  |  |
| Renal failure, acute | 2.1% | 0.0% |  |  |  |  |  |  |  |  |  |  |  |  |
| **Respiratory disorders** |  |  |  |  |  |  |  |  |  |  |  |  |  |  |
| Cough | 0.4% | 0.0% | 0.4% | 0.0% | 0.0% | 0.0% | 0.4% | 0.0% |  |  |  |  |  |  |
| Dyspnoea | 5.0% | 5.3% |  |  | 3.3% | 0.0% | 4.1% | 2.8% |  |  |  |  |  |  |
| Epistaxis |  |  | 1.1% | 0.4% |  |  | 0.8% | 0.9% |  |  |  |  |  |  |
| Oropharyngeal pain | 0.0% | 0.0% | 0.0% | 0.4% |  |  |  |  |  |  |  |  |  |  |
| Pneumonia | 12.0% | 8.5% | 11.3% | 11.2% |  |  | 11.8% | 4.6% | 7.9% | 8.2% | 20.5% | 17.0% | 22.1% | 17.3% |
| **Skin disorders** |  |  |  |  |  |  |  |  |  |  |  |  |  |  |
| Petechiae | 0.8% | 0.0% |  |  |  |  |  |  |  |  |  |  |  |  |
| Pruritus |  |  | 0.8% | 0.0% | 0.0% | 10.0% |  |  |  |  |  |  |  |  |
| Rash | 2.5% | 0.0% | 3.0% | 1.1% | 0.0% | 0.0% | 0.4% | 0.9% | 14.1% | 7.6% |  |  |  |  |
| **Vascular disorders** |  |  |  |  |  |  |  |  |  |  |  |  |  |  |
| Hypertension |  |  | 4.9% | 6.7% |  | 13.3% | 8.1% | 3.7% |  |  |  |  |  |  |
| Hypotension | 3.7% | 2.1% |  |  |  |  | 7.7% | 2.8% |  |  |  |  |  |  |

**Supplementary Table 3.** Percentage of any grade adverse events, classified by System Organ Class and stratified by each study included in the review. Note: Stone et al. did not report adverse events of any grade.

| **Adverse events**  **(≥3 grade)** | **Cortes, 2019** | | **Erba, 2023** | | **Maziarz, 2020** | | **Perl, 2019** | | **Stone, 2017*** | | **Wang, 2022** | | **Wang, 2024** | |
| --- | --- | --- | --- | --- | --- | --- | --- | --- | --- | --- | --- | --- | --- | --- |
|  | **Quizartinib (N=245)** | **Control (N=122)** | **Quizartinib (N=268)** | **Control (N=271)** | **Midostaurin (N=30)** | **Control (N=30)** | **Gilteritinib (N=246)** | **Control (N=109)** | **Midostaurin (N=355)** | **Control (N=354)** | **Gilteritinib (N=73)** | **Control (N=47)** | **Gilteritinib (N=113)** | **Control (N=104)** |
| **Blood and lymphatic system disorders** |  |  |  |  |  |  |  |  |  |  |  |  |  |  |
| Anemia | 36.5% | 31.9% | 10.9% | 7.1% | 20.0% | 23.3% | 62.6% | 34.9% | NA | NA | 31.5% | 34.0% | 77.9% | 64.4% |
| Febrile neutropenia | 33.6% | 27.7% | 44.2% | 42.2% |  |  | 63.0% | 36.7% | NA | NA | 35.6% | 21.3% | 25.7% | 28.8% |
| Leukocytosis | 6.2% | 3.2% |  |  |  |  |  |  | NA | NA |  |  |  |  |
| Leukopenia | 19.5% | 17.0% |  |  |  |  | 21.5% | 17.4% | NA | NA |  |  | 70.8% | 68.3% |
| Neutropenia | 33.6% | 25.5% | 30.6% | 14.6% | 10.0% | 23.3% | 41.9% | 25.7% | NA | NA | 43.8% | 36.2% | 66.4% | 52.9% |
| Pancytopenia | 5.4% | 0.0% |  |  |  |  |  |  | NA | NA |  |  |  |  |
| Thrombocytopenia | 39.0% | 34.0% | 11.3% | 11.2% |  |  | 67.1% | 42.2% | NA | NA | 49.3% | 42.6% | 85.8% | 76.0% |
| **Eye disorders** |  |  |  |  |  |  |  |  |  |  |  |  |  |  |
| Drye eye |  |  |  |  | 20.0% | 16.7% |  |  | NA | NA |  |  |  |  |
| **Gastrointestinal disorders** |  |  |  |  |  |  |  |  |  |  |  |  |  |  |
| Abdominal pain | 22.4% | 17.0% | 28.3% | 23.5% |  |  | 21.5% | 14.7% | NA | NA |  |  |  |  |
| Constipation | 19.5% | 23.4% | 21.1% | 25.7% |  |  | 37.4% | 14.7% | NA | NA | 34.2% | 21.3% |  |  |
| Diarrhea | 29.0% | 36.2% | 37.0% | 35.1% | 23.3% | 40.0% | 45.9% | 29.4% | NA | NA | 38.4% | 17.0% | 28.3% | 24.0% |
| Dyspepsia |  |  | 11.3% | 8.6% |  |  |  |  | NA | NA |  |  |  |  |
| Nausea | 48.1% | 41.5% | 34.0% | 31.3% | 26.7% | 66.7% | 46.7% | 33.0% | NA | NA | 32.9% | 21.3% | 16.8% | 24.0% |
| Stomatitis | 16.6% | 19.1% | 21.5% | 20.9% |  |  | 20.3% | 14.7% | NA | NA |  |  |  |  |
| Vomiting | 33.2% | 21.3% | 24.5% | 19.8% | 23.3% | 73.3% | 27.6% | 13.8% | NA | NA | 24.7% | 17.0% | 19.5% | 23.1% |
| **General disorders** |  |  |  |  |  |  |  |  |  |  |  |  |  |  |
| Asthenia |  |  |  |  |  |  | 19.5% | 9.2% | NA | NA | 23.3% | 14.9% |  |  |
| Fatigue | 39.4% | 28.7% | 10.9% | 8.6% | 30.0% | 26.7% | 34.1% | 12.8% | NA | NA |  |  |  |  |
| Mucositis or stomatitis |  |  |  |  |  |  |  |  | NA | NA |  |  |  |  |
| Oedema peripheral | 21.2% | 23.4% | 11.3% | 13.8% | 30.0% | 26.7% | 29.3% | 11.9% | NA | NA | 21.9% | 10.6% |  |  |
| Pain |  |  |  |  |  |  |  |  | NA | NA |  |  |  |  |
| Pain in extremity |  |  |  |  |  |  | 17.9% | 7.3% | NA | NA |  |  |  |  |
| Pyrexia | 38.2% | 44.7% | 42.3% | 40.7% | 16.7% | 13.3% | 55.7% | 29.4% | NA | NA | 47.9% | 34.0% | 44.2% | 36.5% |
| **Immune system disorders** |  |  |  |  |  |  |  |  |  |  |  |  |  |  |
| Graft-versus-host disease | 12.0% | 0.0% |  |  |  |  |  |  | NA | NA |  |  |  |  |
| **Infections and infestations** |  |  |  |  |  |  |  |  |  |  |  |  |  |  |
| Cellulitis | 6.6% | 0.0% |  |  |  |  |  |  | NA | NA |  |  |  |  |
| Clostridioides difficile  infection | 4.6% | 0.0% |  |  |  |  |  |  | NA | NA |  |  |  |  |
| Device-related infection | 4.6% | 7.4% |  |  |  |  |  |  | NA | NA |  |  |  |  |
| Infection |  |  |  |  |  |  |  |  | NA | NA |  |  |  |  |
| Sepsis or septic shock | 21.6% | 26.6% | 5.7% | 10.4% |  |  |  |  | NA | NA | 5.5% | 10.6% |  |  |
| Upper respiratory tract infection | 8.7% | 0.0% |  |  |  | 30.0% |  |  | NA | NA |  |  |  |  |
| Urinary tract infection | 9.1% | 0.0% |  |  |  |  |  |  | NA | NA |  |  |  |  |
| **Investigations** |  |  |  |  |  |  |  |  |  |  |  |  |  |  |
| ALT increased | 13.3% | 4.3% | 15.8% | 10.1% | 23.3% | 20.0% | 45.9% | 9.2% | NA | NA |  |  | 31.0% | 19.2% |
| AST increased |  |  | 10.6% | 7.1% | 26.7% | 16.7% | 45.5% | 11.9% | NA | NA | 24.7% | 6.4% | 35.4% | 16.3% |
| Blood alkaline  phosphatase increased |  |  |  |  |  |  | 23.6% | 1.8% | NA | NA |  |  |  |  |
| Blood creatine  phosphokinase increased |  |  |  |  |  |  | 26.8% | 3.7% | NA | NA |  |  |  |  |
| Blood lactate dehydrogenase increased |  |  |  |  |  |  |  |  | NA | NA |  |  | 44.2% | 20.2% |
| Electrocardiogram  QT prolonged | 26.6% | 0.0% | 13.6% | 4.1% |  |  |  |  | NA | NA |  |  |  |  |
| Lymphopenia |  |  |  |  |  |  |  |  | NA | NA |  |  | 19.5% | 23.1% |
| Weight decreased | 11.2% | 0.0% |  |  |  |  |  |  | NA | NA |  |  |  |  |
| Weight increased |  |  |  |  |  |  |  |  | NA | NA |  |  | 20.4% | 1.0% |
| **Metabolism and nutrition disorders** |  |  |  |  |  |  |  |  |  |  |  |  |  |  |
| Decreased appetite | 20.3% | 0.0% | 17.4% | 13.4% |  |  | 26.0% | 18.3% | NA | NA | 20.5% | 14.9% |  |  |
| Hyperglycaemia | 6.2% | 7.4% |  |  |  |  | 20.3% | 12.8% | NA | NA |  |  | 24.8% | 14.4% |
| Hypoalbuminemia |  |  |  |  |  |  | 15.9% | 6.4% | NA | NA |  |  |  |  |
| Hypocalcemia | 12.0% | 10.6% | 9.8% | 10.8% |  |  | 21.5% | 5.5% | NA | NA |  |  | 26.5% | 21.2% |
| Hypokalemia | 32.4% | 27.7% | 35.1% | 35.8% |  |  | 42.7% | 31.2% | NA | NA | 21.9% | 21.3% | 40.7% | 38.5% |
| Hypomagnesemia | 15.4% | 0.0% | 11.3% | 11.2% |  |  | 20.7% | 11.0% | NA | NA |  |  |  |  |
| Hyponatremia | 9.1% | 0.0% |  |  |  |  | 15.9% | 5.5% | NA | NA | 19.2% | 6.4% | 23.0% | 20.2% |
| Hypophosphatemia | 10.0% | 10.6% | 10.2% | 9.0% |  |  | 18.7% | 4.6% | NA | NA |  |  |  |  |
| **Muscoloskeletal disorders** |  |  |  |  |  |  |  |  |  |  |  |  |  |  |
| Arthralgia |  |  | 10.9% | 13.1% | 20.0% | 10.0% | 13.8% | 5.5% | NA | NA |  |  |  |  |
| Back pain |  |  | 7.2% | 10.4% |  |  | 17.1% | 11.9% | NA | NA |  |  |  |  |
| Myalgia | 37.8% | 28.7% |  |  |  |  | 15.9% | 3.7% | NA | NA |  |  |  |  |
| **Neoplasms** |  |  |  |  |  |  |  |  |  |  |  |  |  |  |
| Acute myeloid leukemia |  |  |  |  |  |  | 15.0% | 3.7% | NA | NA |  |  |  |  |
| **Nervous system disorders** |  |  |  |  |  |  |  |  |  |  |  |  |  |  |
| Dizziness | 14.9% | 10.6% |  |  | 20.0% | 10.0% | 20.3% | 1.8% | NA | NA |  |  |  |  |
| Dysgeusia |  |  |  |  |  |  | 12.2% | 4.6% | NA | NA |  |  |  |  |
| Headache | 21.6% | 17.0% | 27.5% | 19.8% | 23.3% | 26.7% | 32.5% | 14.7% | NA | NA |  |  |  |  |
| Syncope | 4.6% | 0.0% |  |  |  |  |  |  | NA | NA |  |  |  |  |
| Tremor |  |  |  |  | 13.3% | 23.3% |  |  | NA | NA |  |  |  |  |
| **Psychiatric disorders** |  |  |  |  |  |  |  |  |  |  |  |  |  |  |
| Insomnia |  |  | 14.0% | 11.2% | 20.0% | 13.3% | 18.7% | 5.5% | NA | NA |  |  |  |  |
| **Renal and urinary disorders** |  |  |  |  |  |  |  |  |  |  |  |  |  |  |
| Renal failure, acute | 5.8% | 0.0% |  |  |  |  |  |  | NA | NA |  |  |  |  |
| **Respiratory disorders** |  |  |  |  |  |  |  |  |  |  |  |  |  |  |
| Cough | 23.2% | 13.8% | 18.9% | 16.4% | 20.0% | 26.7% | 33.7% | 10.1% | NA | NA |  |  |  |  |
| Dyspnoea | 20.3% | 8.5% |  |  | 23.3% | 10.0% | 26.4% | 6.4% | NA | NA |  |  |  |  |
| Epistaxis |  |  | 15.1% | 10.8% |  |  | 20.3% | 7.3% | NA | NA |  |  |  |  |
| Oropharyngeal pain | 10.4% | 0.0% | 10.2% | 6.7% |  |  |  |  | NA | NA |  |  |  |  |
| Pneumonia | 15.8% | 10.6% | 14.7% | 15.3% |  |  | 20.7% | 7.3% | NA | NA | 26.0% | 17.0% | 29.2% | 26.0% |
| **Skin disorders** |  |  |  |  |  |  |  |  |  |  |  |  |  |  |
| Petechiae | 11.2% | 0.0% |  |  |  |  |  |  | NA | NA |  |  |  |  |
| Pruritus |  |  | 13.2% | 14.9% | 20.0% | 23.3% |  |  | NA | NA |  |  |  |  |
| Rash | 22.8% | 18.1% | 26.0% | 24.6% | 20.0% | 20.0% | 18.7% | 9.2% | NA | NA |  |  |  |  |
| **Vascular disorders** |  |  |  |  |  |  |  |  |  |  |  |  |  |  |
| Hypertension |  |  | 10.9% | 12.3% |  | 30.0% | 17.9% | 9.2% | NA | NA |  |  |  |  |
| Hypotension | 13.3% | 10.6% |  |  |  |  | 20.7% | 7.3% | NA | NA |  |  |  |  |


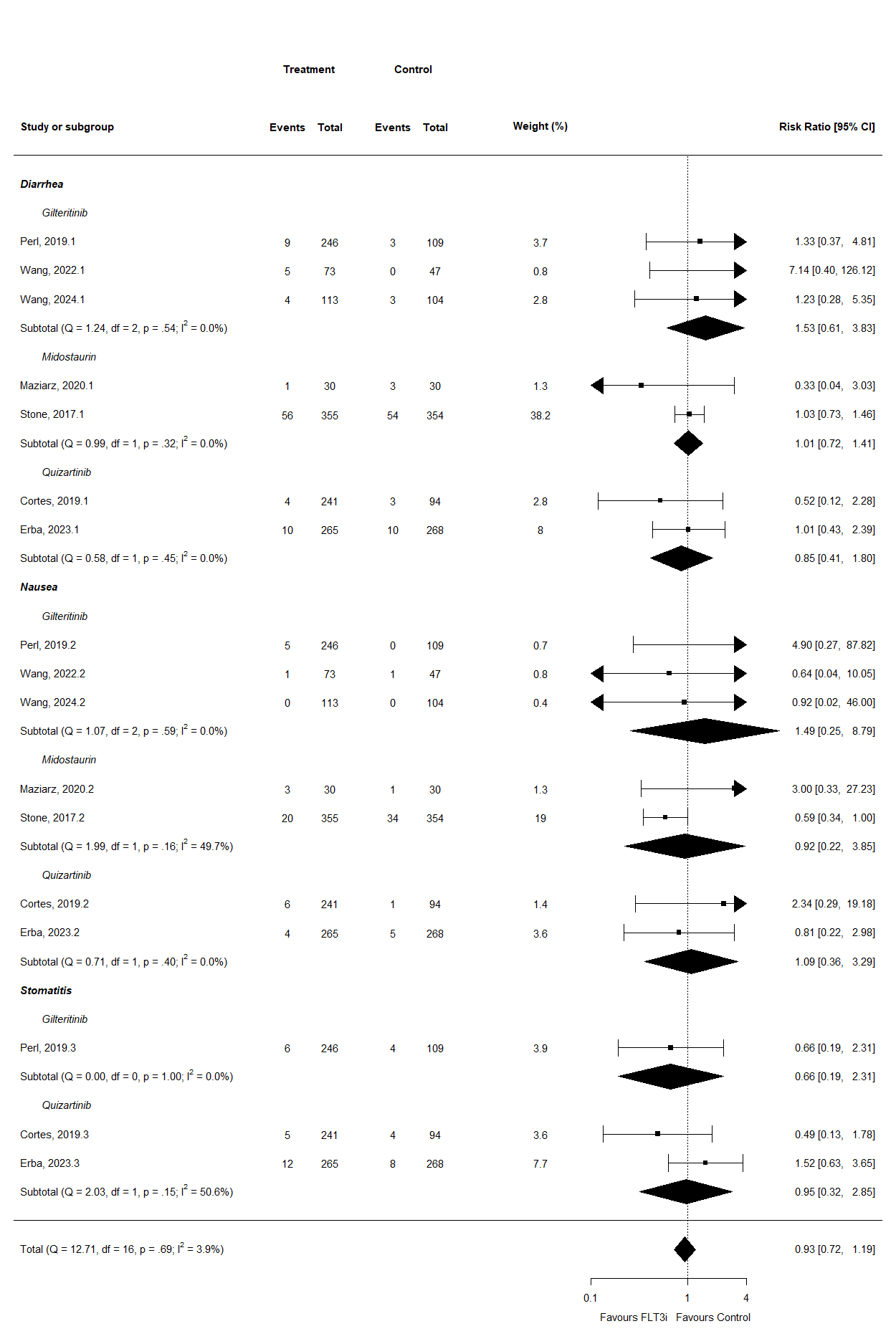
**Supplementary Figure 3.** Forest plot for adverse events related to gastrointestinal disorders.


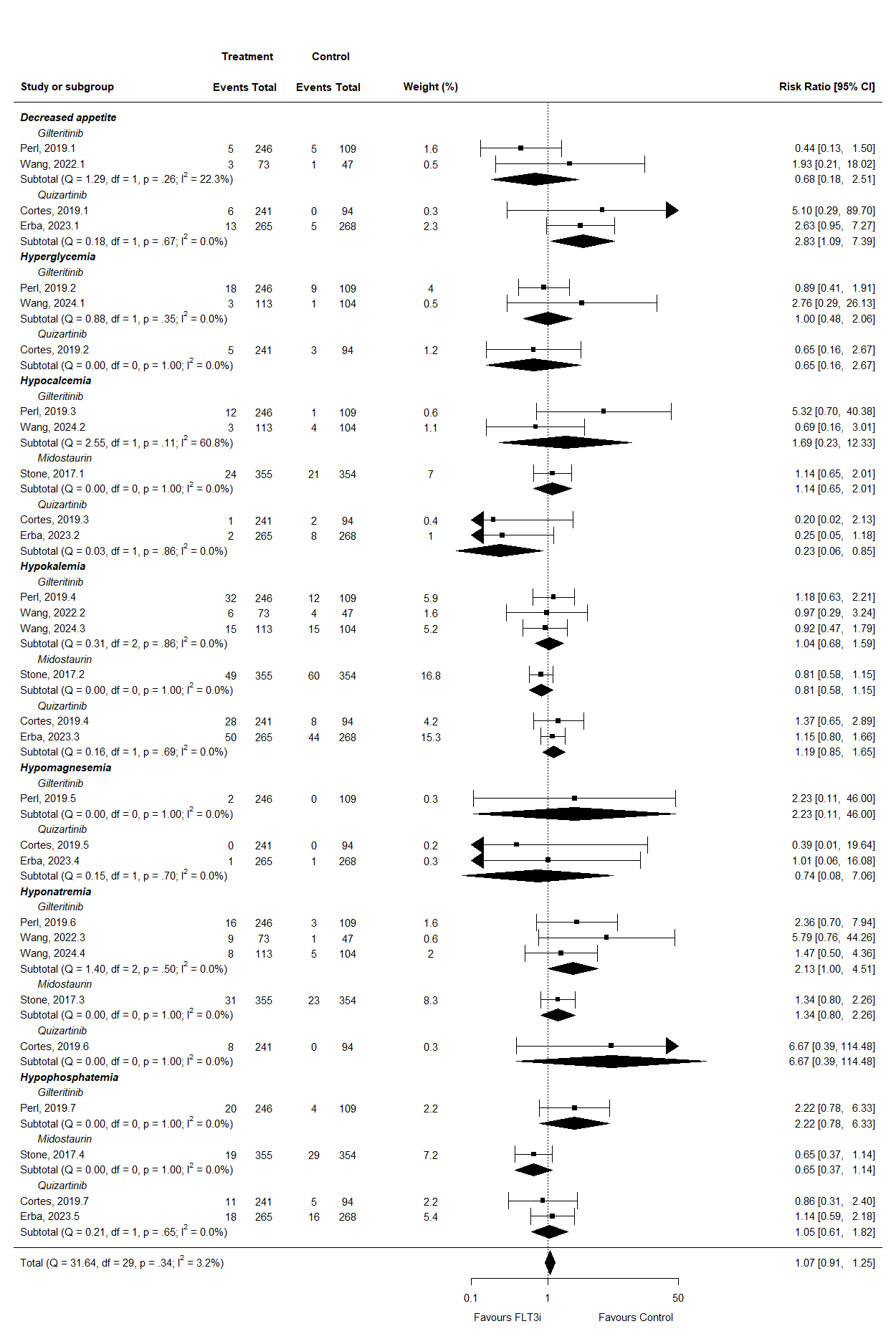


**Supplementary Figure 4.** Forest plot for adverse events related to metabolism disorders.


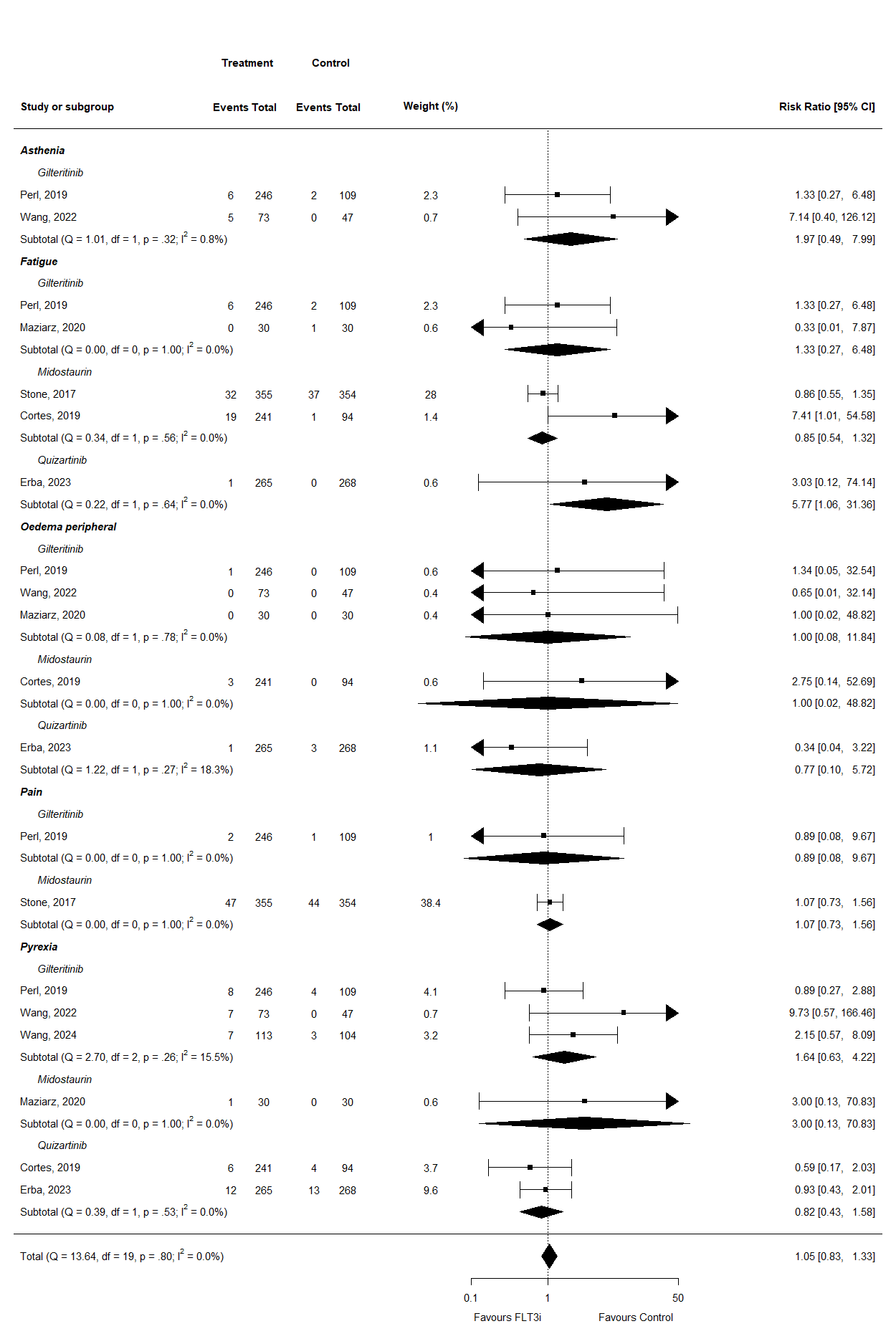
**Supplementary Figure 5.** Forest plot for adverse events related to general disorders.


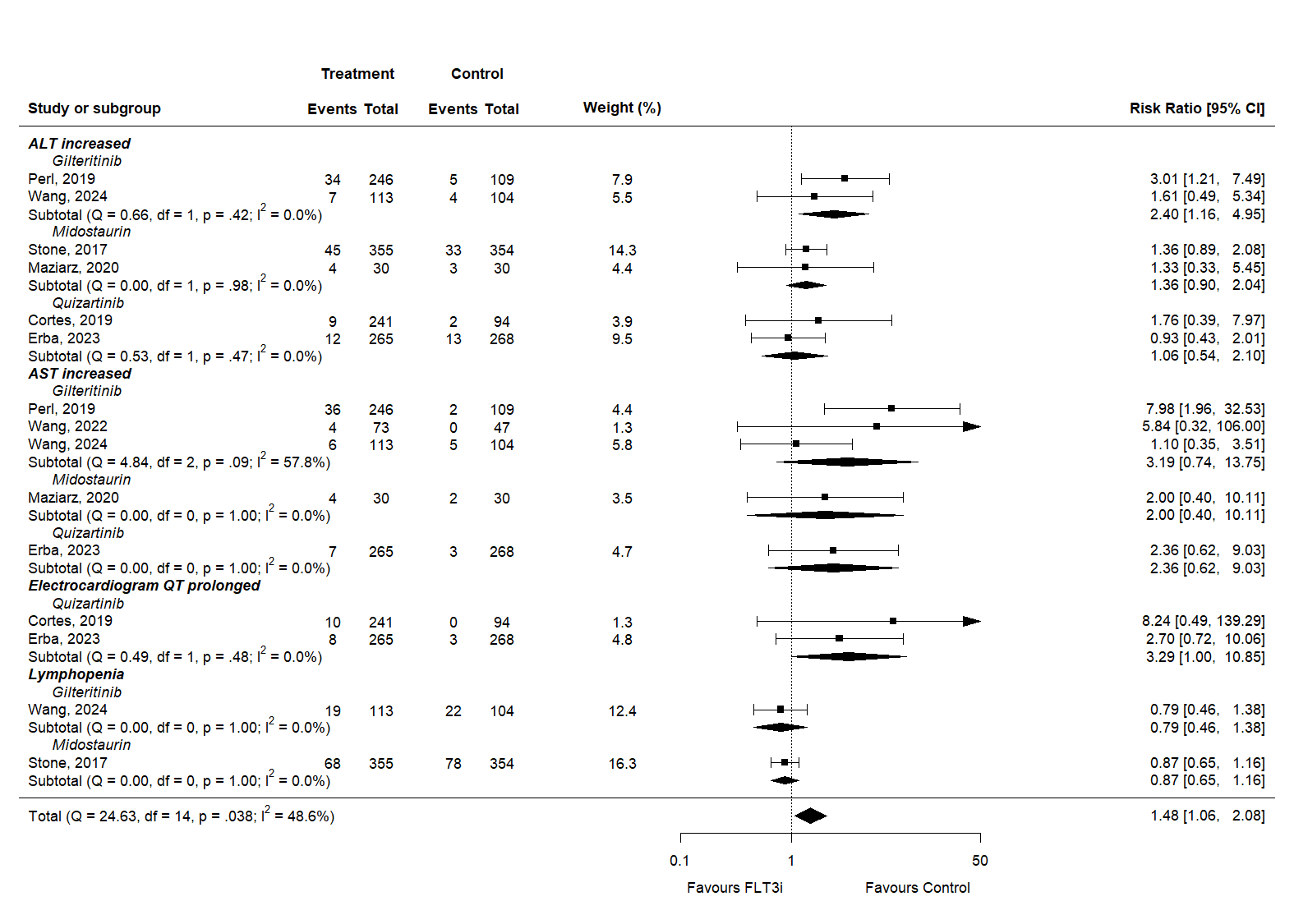


**Supplementary Figure 6.** Forest plot for adverse events related to investigation.


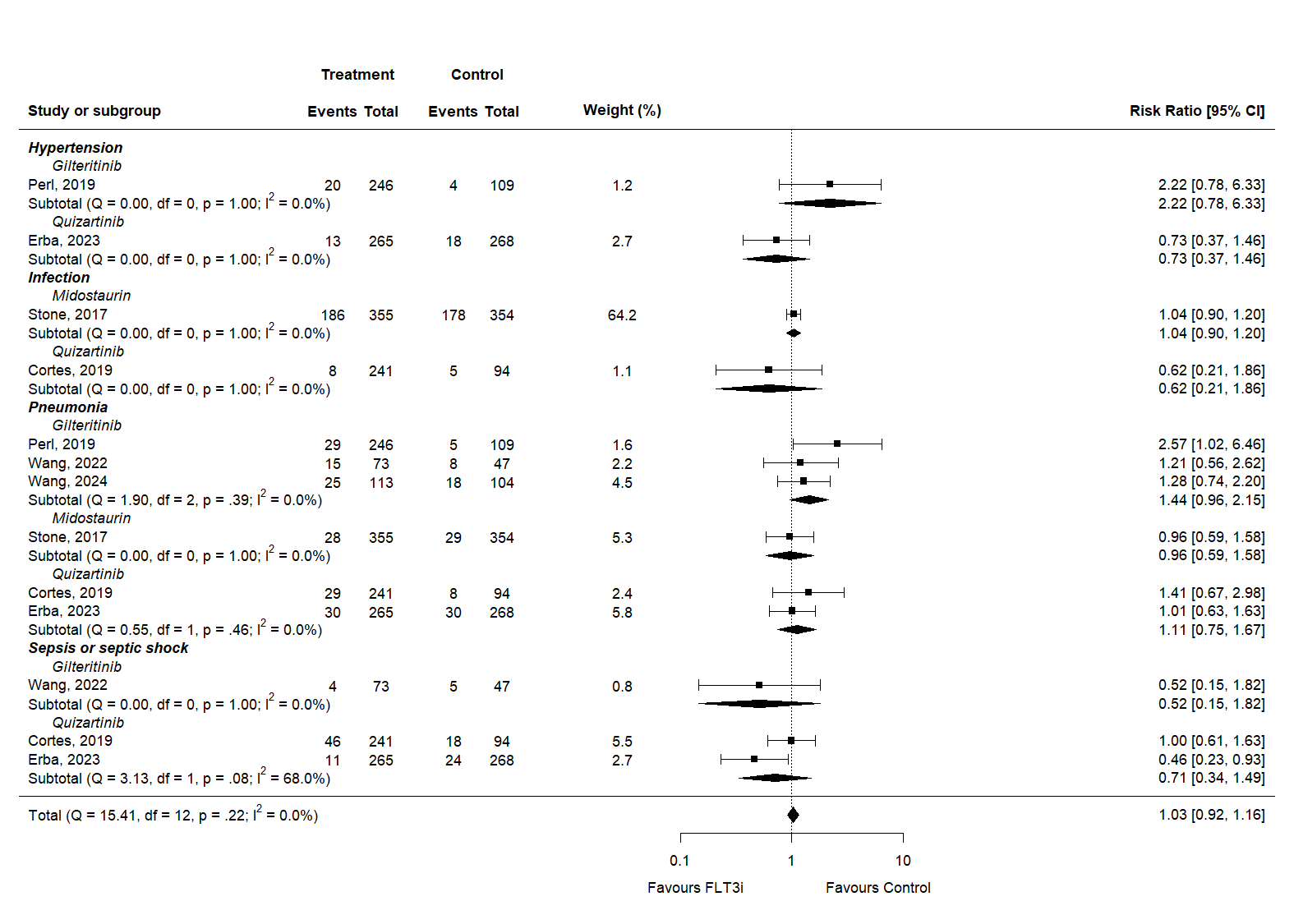


**Supplementary Figure 7.** Forest plot for other adverse events.
